# Accurate Measurement of 5-Methylcytosine and 5-Hydroxymethylcytosine in Human Cerebellum DNA by Oxidative Bisulfite on an Array (OxBS-Array)

**DOI:** 10.1371/journal.pone.0118202

**Published:** 2015-02-23

**Authors:** Sarah F. Field, Dario Beraldi, Martin Bachman, Sabrina K. Stewart, Stephan Beck, Shankar Balasubramanian

**Affiliations:** 1 Cancer Research UK Cambridge Institute, University of Cambridge, Li Ka Shing Centre, Robinson Way, Cambridge, CB2 0RE, United Kingdom; 2 Department of Cancer Biology, UCL Cancer Institute, University College London, London WC1E 6BT, United Kingdom; 3 Department of Chemistry, University of Cambridge, Cambridge, CB2 1EW, United Kingdom; Bellvitge Biomedical Research Institute (IDIBELL), SPAIN

## Abstract

The Infinium 450K Methylation array is an established tool for measuring methylation. However, the bisulfite (BS) reaction commonly used with the 450K array cannot distinguish between 5-methylcytosine (5mC) and 5-hydroxymethylcytosine (5hmC). The oxidative-bisulfite assay disambiguates 5mC and 5hmC. We describe the use of oxBS in conjunction with the 450K array (oxBS-array) to analyse 5hmC/5mC in cerebellum DNA. The “methylation” level derived by the BS reaction is the combined level of 5mC and 5hmC at a given base, while the oxBS reaction gives the level of 5mC alone. The level of 5hmC is derived by subtracting the oxBS level from the BS level. Here we present an analysis method that distinguishes genuine positive levels of 5hmC at levels as low as 3%. We performed four replicates of the same sample of cerebellum and found a high level of reproducibility (average r for BS = 98.3, and average r for oxBS = 96.8). In total, 114,734 probes showed a significant positive measurement for 5hmC. The range at which we were able to distinguish 5hmC occupancy was between 3% and 42%. In order to investigate the effects of multiple replicates on 5hmC detection we also simulated fewer replicates and found that decreasing the number of replicates to two reduced the number of positive probes identified by > 50%. We validated our results using qPCR in conjunction with glucosylation of 5hmC sites followed by MspI digestion and we found good concordance with the array estimates (r = 0.94). This experiment provides a map of 5hmC in the cerebellum and a robust dataset for use as a standard in future 5hmC analyses. We also provide a novel method for validating the presence of 5hmC at low levels, and highlight some of the pitfalls associated with measuring 5hmC and 5mC.

## Introduction

DNA methylation has a crucial role in gene expression and thus in differentiation and development[1]. In light of this many tools have been developed to investigate methylation patterns across the genome[2]. However, recently it has been discovered that there are several different types of base modifications in mammalian DNA, which have previously been indistinguishable. The first type of modification identified was 5-methylcytosine (5mC)[3], but recently other modifications have been found which include: 5-hydroxymethylcytosine (5hmC), 5-formylcytosine (5fC) and 5-carboxylcytosine (5caC)[4–6]. Proposed models of demethylation suggest that there may be two pathways; either it may occur passively, during DNA replication, or actively through enzymatic action[7–9]. The TET enzymes catalyse the sequential oxidation of 5mC to 5hmC, then 5fC and finally 5caC [6,7]. It has been proposed that this TET mediated oxidation can ultimately lead to demethylation via base excision repair of one or more oxidised intermediates via a process involving thymidine glycosylase (TDG) or other repair enzymes[10].

It also remains to be addressed whether 5hmC, 5fC and 5caC are functional modifications in their own right, as opposed to simply being intermediates of active demethylation. Patterns of 5hmC distribution have been found to vary from 5mC distribution both during development and in adult cells[10–12]. Changes in 5hmC have been associated with adult onset diseases including hypertension and Alzheimer’s, and with neuronal maintenance[13–15]. Functional studies of the binding domain of MeCP2 (a transcription factor implicated in Rett syndrome), have shown that mutations associated with the disease preferentially impact binding to 5hmC, as opposed to 5mC[16].

To investigate the role of 5mC and 5hmC it is necessary to be able to accurately detect and quantitate the levels of these modifications at single base resolution. The most common method for interrogating DNA methylation, at the base pair level, is to react the DNA with bisulfite (BS). BS treatment deaminates C bases to uracil, which are read as thymines (T) in downstream assays[17,18]. Both 5mC and 5hmC are resistant to deamination by BS and are read as C in downstream assays [17,18]. Thus, the untransformed C bases quantified at single base resolution by BS treatment actually represent the sum of 5mC and 5hmC levels at that base. Our laboratory developed an oxidative bisulfite (oxBS) treatment that allows the disambiguation of 5mC from 5hmC[19,20]. It was observed that 5fC is deaminated to uracil by bisulfite treatment; therefore, by selectively oxidising 5hmC to 5fC prior to bisulfite treatment, only 5mC remains unconverted by bisulfite treatment. By comparing the results of sequencing or array probes for a BS and oxBS treated sample it becomes possible to accurately quantify both 5mC and 5hmC.

The Infinium 450K methylation array targets cytosine-phosphate-guanine (CpG) nucleotides (although non-CpG sites are also included), in conjunction with BS treatment, it can be used to interrogate methylation at > 450,000 sites across the human genome[2]. Many studies have made use of this technology and the array has been validated by both targeted and whole genome sequencing[2]. In order to quantify both 5mC and 5hmC at these sites we treated the sample both with BS and with oxBS. The methylation level, as measured by untransformed C, on the oxBS treated sample is the true level of 5mC, while the level measured on the BS treated sample is the actual level of 5mC plus 5hmC, therefore, by subtracting the oxBS level from the BS level we were able to ascertain the level of 5hmC alone.

In general, the levels of total 5hmC observed across the genome are approximately 10 fold lower than those of 5mC, although these levels vary across tissue types[21]. In our sample the levels of 5mC, as measured by liquid chromatography-mass spectrometry (LC-MS), were four fold higher than the levels of 5hmC. At individual sites the level of 5mC may be considerably higher than the level of 5hmC resulting in a small difference between untransformed C measured by BS and by oxBS. Thus when doing the BS—oxBS subtraction it is imperative to be able to distinguish between a genuine difference, resulting from the presence of 5hmC, and the noise inherent in the assay. Our goals in this experiment are to evaluate the reproducibility of this method, to validate the levels of 5hmC measured, and to determine the lower limit of 5hmC detectability empirically, rather than by choosing an arbitrary cut off point. To this end, we have chosen a single, commercially available sample of cerebellum DNA since the highest levels of 5hmC in mammals are observed in adult neuronal tissues[10,21]. This sample might be used as a control in future investigations of 5hmC (GEO accession: GSE63179).

## Results

**Fig 1:**
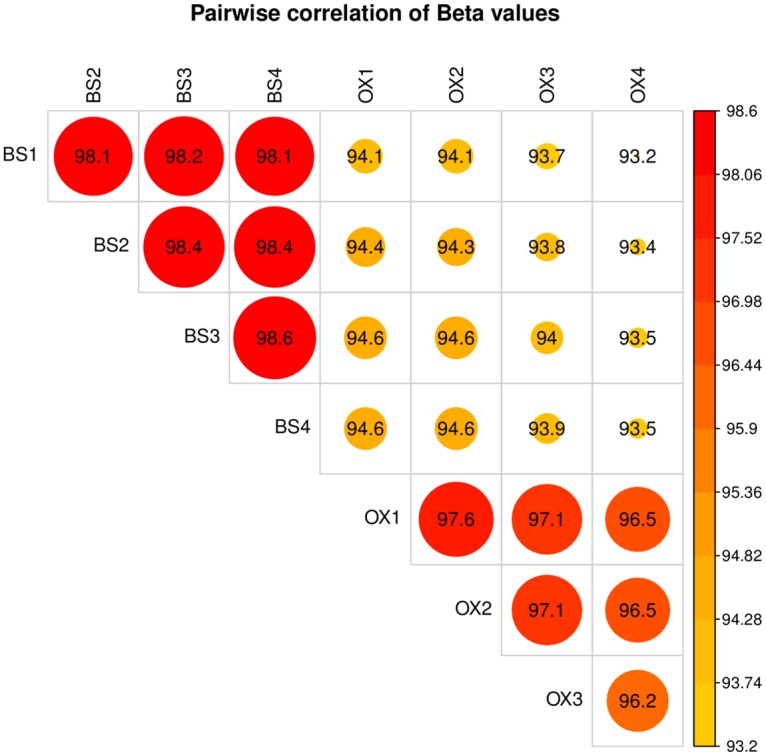
Pairwise correlation in beta values between BS and oxBS arrays. The same sample was divided into four BS and four oxBS replicates. Beta values are generated by the minfi analysis package and represent the percentage of unconverted C’s at any given locus. The average correlation of BS replicates is 98.3 and of the oxBS replicates is 96.8. The average correlation between the two methods is 94.0.

**Fig 2:**
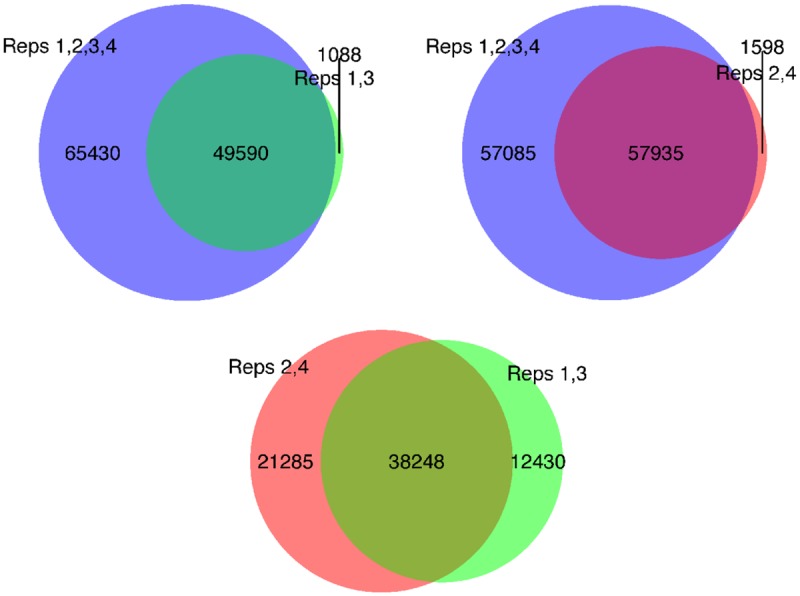
Effect of number of replicates on 5hmC detection. Top left: Number of probes detected as differentially methylated, i.e. as hydroxymethylated (having a positive 5hmC signal), with all four replicates and with only replicates 1 and 3. Top right: Using only replicates 2 and 4. The number of probes observed in two replicates but not in four fall within the expected 1% FDR. Bottom: Probes with 5hmC in pair 1 and 3 compared to pair 2 and 4. Only 38,246 probes were observed in both pairs.

We performed four replicates of BS and oxBS, which showed a high degree of reproducibility, with the beta values for BS replicates having an average correlation coefficient (r) of 98.3 (98.1–98.6), and the oxBS replicates having an average r of 96.8 (96.2–97.6). Comparing the beta values for BS replicates to the oxBS replicates gave and average r of 94.0 (range: 93.4–94.6; for the distribution of beta values see S1 Fig.; for pairwise correlations see Fig. 1 and S2 Fig.). Principal components analysis and hierarchical clustering of the arrays showed a clear separation of BS and oxBS treated samples with no clear outliers (see S1 Document and S2 and S3 Figs.). In our analysis we measured 5mC and 5hmC in all four replicates and then used the average of these replicates as our final measurement of 5mC and 5hmC. In this paper we refer to probes as “positive” where we reliably observe 5hmC at any level above 0%. In total 114,734 probes showed a significant positive measurement for 5hmC at a false discovery rate (FDR) <0.01 (see S5 and S6 Figs.). In order to investigate the effects of multiple replicates on 5hmC detection we also simulated fewer replicates by analysing replicates 1 and 3 separately from replicates 2 and 4. For replicates 1 and 3 we were able to detect 50,678 probes with significant difference between BS and oxBS (either positive or negative), while for replicates 2 and 4 we were able to detect 59,533 probes. The overlap between the probe subsets was 38,248 probes. In addition it should be noted that analysing these subsets yielded probes which did not pass the significance test when all four replicates were analysed together; 1,088 in the 1 and 3 subset and 1,598 in the 2 and 4 subset (see Fig. 2). These values are consistent with the 1% FDR threshold. The median difference in effect size between BS and oxBS in four replicates is 14.0% and between two replicates is 19.4 and 19.8% respectively (see S6 Fig.).

**Fig 3:**
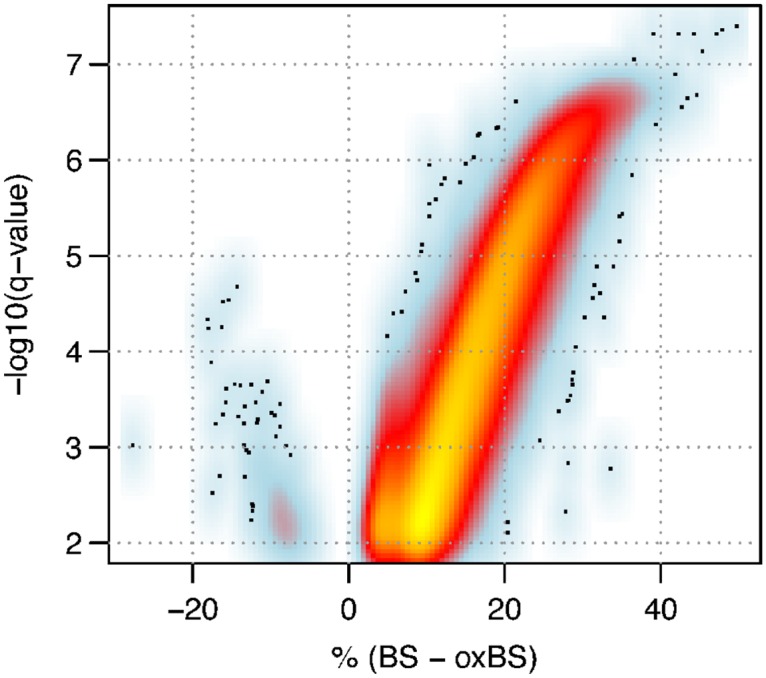
Distribution of 5hmC levels. Only probes which were significantly differentially methylated between BS and oxBS are shown hence the lack of probes around zero. For the plot of all probes see S5 Fig. Negative values fall within the expected 1% FDR.

At any given site a mixture of states will be observed, with some molecules methylated, hydroxymethylated or unmethylated. The levels of 5hmC we observe, at any given C, fall between 2.5% and 49.7% with the median at 14.1%. In the replicate subsets the lowest limits were 4.3% and 4.2% respectively (see S7 and S8 Figs.). It should be noted that the detection of 5hmC relies on the difference between BS and oxBS read-outs, therefore it is expected that at sites with zero or low levels of 5hmC this difference may be negative as a consequence of inevitable random noise. In our data we do, indeed, see “negative” values that pass the threshold for significance. However, the number of these “negative” calls also falls within the 1% FDR expected (see Fig. 3 and S6 Fig., see S4 Fig. for distribution of p-values in positive and negative differences between BS and oxBS).

**Fig 4:**
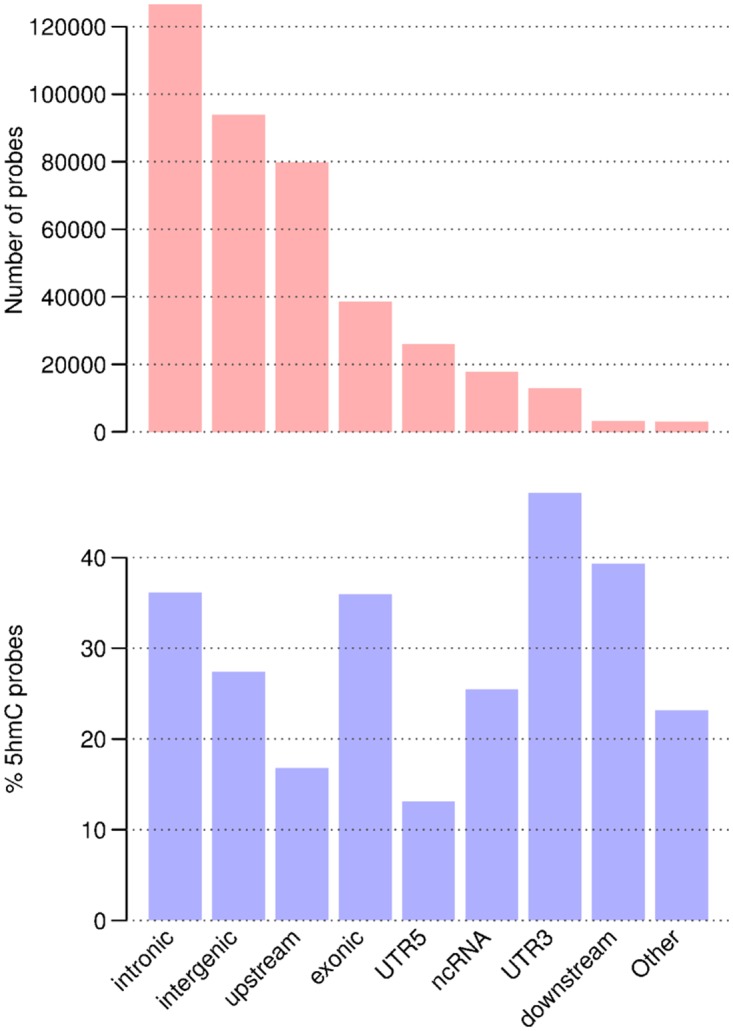
Top: the number of probes in each genomic region in the entire 450K array. The highest number of probes are in introns, followed by intergenic regions. Bottom: the proportion of probes in each region found to have a positive 5hmC signal. While only a small number of probes are designed in 3’UTR’s (12984), this region has the highest proportion of probes with a positive 5hmC signal (47%). Regions labeled “Other” are those that do not fit into any of the previous categories.

**Fig 5:**
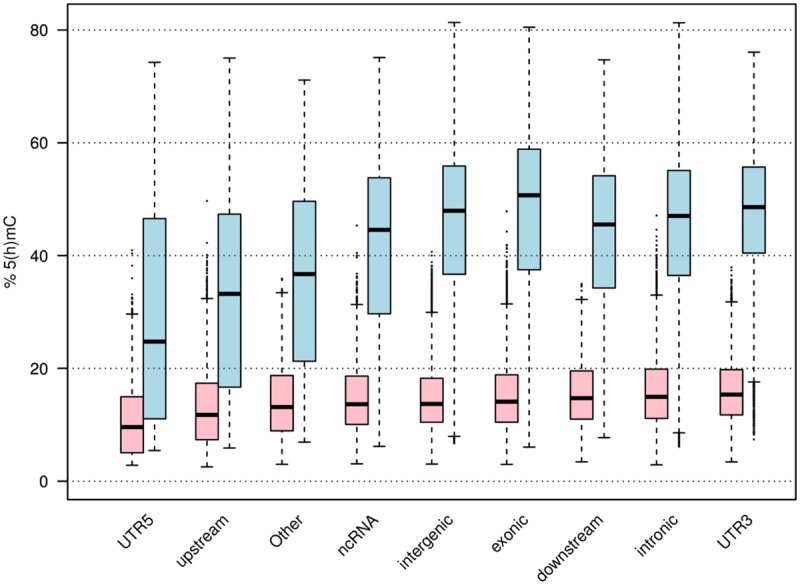
Distribution of 5mC (blue) and 5hmC (pink) levels across regions. The lowest median levels of both are in 5’UTR’s, however, beyond that there is little correlation between 5mC and 5hmC levels. Median 5hmC levels are similar across all regions, while 5mC levels show greater variation.

**Fig 6:**
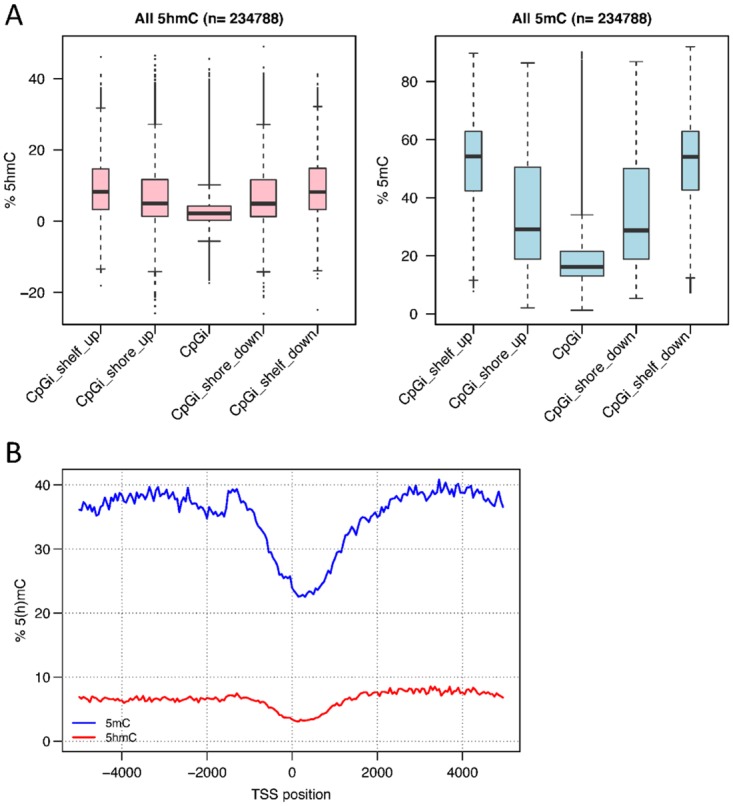
Genomic distribution of 5mC and 5hmC. 6a: Distribution of 5hmC and 5mC levels at CpG islands, shores (0–2 kb) and shelves (2–4 kb). 5mC levels show a steep rise moving away from CpG sites, 5hmC levels are lowest at CpG sites but show less variation between shores and shelves. The width of the boxes is proportional to the number of underlying probes. 6b: Distribution of 5mC and 5hmC levels around the TSS. Levels of both types of methylation rise steeply moving away from TSS’s.

**Fig 7:**
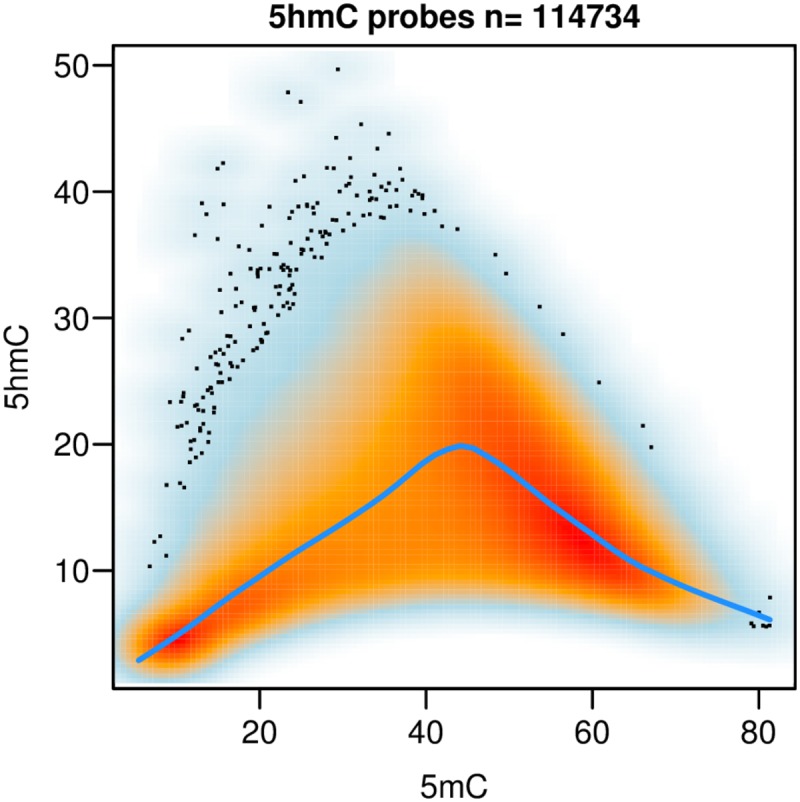
Scatter plot of 5hmC vs 5mC. 5hmC levels are highest at intermediate levels of 5mC (~40%).

We measured global levels of 5mC and 5hmC in the sample by LC-MC; 5mC was found at 4.6% (averaged from three replicates: 4.24, 4.55 and 5.19%) of all Cs, while 5hmC was found at 1.14% (averaged from three replicates: 1.02, 1.10 and 1.25%). The distribution of positive 5hmC probes was between 13% and 47% of all 450K array probes for any given region, with the highest number of probes observed at downstream, exonic and intronic regions. Here upstream and downstream regions were defined as 1 kb from the transcription start site (TSS) and transcription termination site of each gene, respectively. The lowest number of positive probes was observed in 5’UTRs (13%; see Fig. 4). The average levels of 5hmC were quite similar across all genomic regions (mean 14%, Fig. 5). The lowest average levels were found upstream of genes and in the 5’UTRs (mean 10%), this correlates with the low percentage of probes in this region found to have a positive 5hmC signal. These regions also showed the lowest levels of 5mC (see Fig. 5). The highest average 5hmC levels were found in introns and 3’UTRs. However, at individual Cs, levels of 5hmC as high as 42% were observed in gene bodies (particularly in introns) (see Fig. 5). The highest average levels of 5mC were observed in exons (see Fig. 5). Average levels of both 5hmC and 5mC were lower at CpG islands than at the CpG shores and shelves (regions flanking CpG islands by 0 to 2 kb, and 2 to 4 kb respectively, Fig. 6a). Transcription start sites also show a marked decrease in 5mC and 5hmC levels, which rise steeply as one moves away from them (see Fig. 6b). These regional levels correlate with those found in other studies and by other methods[22] (see S10 Fig.). In general the highest levels of 5hmC occurred where 5mC was at ~ 50%, and there was a greater correlation of 5mC-5hmC levels at the lowest levels of 5mC and 5hmC than at the highest (See Fig. 7). GO analysis did not reveal any particular enrichment of terms associated with 5hmC positive probes in this sample.

**Fig 8:**
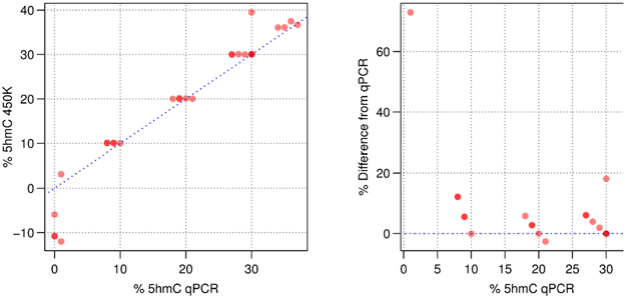
Comparison between oxBS arrays and qPCR. Right: Correlation between 5hmC detected by qPCR vs array probes. Left: Discrepancy between qPCR and arrays quantified as percentage difference relative to qPCR (See also S1 Document). Data points having the same coordinates appear in darker red.

We validated these results using an orthogonal assay that employs glucosylation of 5hmC sites followed by MspI digestion, which is specific for CCGG sites, and qPCR. 5hmC glucosyltransferase can be used to modify 5hmC, yielding glucosyl-5hmC, which is not susceptible to digestion by MspI[23]. Therefore by performing qPCR on glucosylated, unglucosylated and undigested DNA it is possible to ascertain the levels of 5hmC at a given site. We chose 27 sites that had a range of 5hmC values according to the oxBS array data from 0% to 40% and including apparently “negative” values. Sites were selected first by 5hmC level then by the availability of a CCGG motif at that site (see S3 Table). The correlation coefficient between the 450K results and the qPCR results was 0.94. Sites with negative values by the oxBS array clustered around zero on the qPCR (See Fig. 8 and S2 Table) demonstrating that these “negative” values are indeed an artefact resulting from the random distribution around 0. The correlation between the oxBS array and the qPCR validation was maintained across the dynamic range of the array experiment from 3% to 40% (see Fig. 8). Two recently published studies have also measured 5hmC in brain tissue using arrays. Stewart et al. [24] used the oxBS array assay while Chopra et al. [22] used TAB array. Our data shows consistency with the dataset from Stewart et al. (see S10 Fig.) whereas the consistency was less good with the TAB array data, suggesting the possibility of method-based differences (see S9 Fig.). However, we must be cautious in drawing conclusions about the comparisons, as the samples were different. The independent validation of our method by a distinct assay (glucosylation and MspI digestion) across the full dynamic range of our data, provides us with confidence in the accuracy of our measurements.

## Discussion

In this paper we have presented 5hmC and 5mC data for a single, commercially available, cerebellum DNA sample. We have also presented an analysis technique that allows validation of a genuinely positive 5hmC signal at low levels. We have demonstrated the value of multiple replicates. While valuable information can be gained from fewer than four replicates, researchers should be aware of the level of information that may be missed. We found that four replicates allowed the identification of 98%–132% more 5hmC positive probes than only two replicates. It must be stressed that with the methylation array, as with expression arrays, a lack of positive signal cannot be interpreted to mean that there is no 5hmC at that position; only that either the level of 5hmC is below the detection threshold, or that the call quality of the assay at that site was insufficient. However, given that we observed (and validated) 5hmC levels below 5% it is likely that probes with levels below the detection threshold are very close to zero. The amount of 5hmC at any given probe was estimated by subtracting the level of methylation detected in the oxBS arrays, i.e. 5mC only, from the methylation in the BS arrays, i.e. sum of 5mC and 5hmC. This approach does not explicitly consider that the percentage of C, 5mC and 5hmC must sum to 1. Future work aimed at modeling this constraint would be beneficial to provide more robust estimates of cytosine modification.

The aim of demonstrating the accuracy, and highlighting the pitfalls, of this method can be met using a single sample with multiple replicates, in order to answer the biological question of the role of 5hmC many more samples will be required. Alternative methods have and are being developed[22,24] In every sequencing experiment a trade-off must be made between the number of sites interrogated and the depth of sequencing. In methylation experiments this trade-off becomes even more pertinent as the percentage of modified C’s at any given site may be very low (as we have observed in this study), in order to accurately call a site with 10% 5hmC a sequencing depth of hundreds of reads would be required. The 450K array is therefore a particularly valuable tool for case/control regional association studies, as it provides wide coverage and can accurately call 5hmC levels <5%. By making the data for this commercial sample available we hope to provide the field with a resource in the form of a control sample that may be analysed alongside experimental samples. This control would permit greater confidence of 5hmC calls and give an estimate of the variance of the particular assay used.

## Methods

We used a single sample of cerebellum DNA from a 25 year old, healthy male (amsbio ltd. Catalogue number: D1234039-DC, Lot No: A807003). The DNA was taken from fresh tissue. In order to measure the reproducibility of the method, this sample was split into eight 1.2 kg aliquots, and each aliquot was separately treated with the CEGX BS or oxBS reagents according to the manufacturers specifications (http://www.cambridge-epigenetix.com/uploads/files/TrueMethyl24_UGuide.pdf). The DNA was first sheared (using Covaris G tubes) to 10 kb, and then purified on P6 columns (New England Biolabs). Four of the aliquots were subjected to oxidation and four to mock oxidation prior to the BS treatment (see CEGX protocol). Following the BS reaction the samples were re-quantified. The recovered volume was between 12 and 18 μl, and the concentration was between 30 and 60 ng/μl for the oxBS treated aliquots and between 40 and 60 ng/μl for the BS treated aliquots. Since we started with 1.2 Kg, the average loss of material (as measured by Qubit after bisulfite treatment, according to the manufacturers specifications) for oxBS was 44% and for BS was 38%. The samples were then run on the Infinium 450K chip by Genome Services at the Department of Pathology (Cambridge University). The only modification to the standard 450K loading protocol was that 7 Ϗl of sample were loaded with 1 μl NaOH, as opposed to the standard protocol which calls for 4 μl of sample and 4 μl of NaOH; in this way we ensured that a minimum of 210 ng of sample were loaded on to each array.

Total levels of 5hmC and 5mC in the sample were measured by LC-MS. Global levels of mC and hmC were analyzed as described in Booth *et al*.[19] but using a Q-Exactive mass spectrometer (Thermo). The levels are expressed as percentage of total cytosines[19]. The array results were validated by glycosylation followed by qPCR using the Zymo Quest 5-hmC detection kit and the Zymo QuestTaq qPCR PreMix respectively according to the manufacturers specifications (http://www.zymoresearch.com/downloads/dl/file/id/126/e2026i.pdf).

### Analysis

The raw idat files produced by the Illumina platform were analysed with the R/Bioconductor package minfi[25]. The applied normalization method was the subset-quantile within array normalisation (SWAN)[26]. Probe qualities within each array were assessed using the function detectionP in the minfi package and probes with detection p-value < 0.01 in more than one array were excluded from further analysis. Probes differentially methylated between BS and oxBS were identified by F-test with variance shrinkage fitting the treatment “BS” or “oxBS” as categorical variable (function dmpFinder with shrinkVar = TRUE). Raw p-values were converted to false discovery rate (FDR) to control for multiple testing and probes with FDR < 0.01 were deemed as significantly differentially methylated. The beta values were used as methylation percentage to interpret the data. To summarize methylation of each probe within BS and within oxBS, the average of beta values of the 4 replicates was taken. S1 Document includes full details of the analyses and should enable the reader to reproduce the findings presented here (see also http://github.com/dariober/Illumina-450K-oxBS for the code in the S1 Document).

### Ethics statement

This DNA sample was collected under the BioChain Institute Inc. Institutional Review Board (IRB). This IRB is registered with the Office for Human Research Protections (OHRP), registration number IRB00008283 and has been issued with Federal Wide Assurance (FWA), FWA00017355, for the Protection of Human Subjects for Institutions within the United States by OHRP of the Department of Health and Human Service (DHHS), USA.

## Supporting Information

S1 Document (PDF)

S1 Fig Density plot of beta values after having removed failed probes and after SWAN normalization. (TIFF)

S2 Fig Principal components analysis based on probe beta values. In blue the “BS” arrays, in red the “oxBS” arrays.
(TIFF)

S3 Fig Hierarchical clustering of arrays on beta values. (TIFF)

S4 Fig Histogram of pvalues for the evidence of BS—oxBS <0 (grey bars) and BS—oxBS >0 (red bars). (TIFF)

S5 Fig Probes differentially methylated between BS and oxBS arrays. Left: All the probes passing quality control (n = 401866). Right: Probes passing quality control and with FDR <0.01 for difference between BS and oxBS (n = 115020, n positive = 114734).
(TIFF)

S6 Fig Effect size, i.e. significant difference in beta values between BS and oxBS, detected with four and two replicates. (TIFF)

S7 Fig Difference between BS and oxBS beta values for probes detected significantly different in both the 4 replicates and in replicates 1 and 3. The shaded grey band marks the diagonal plus and minus 5, i.e. probes outside the grey band differ between 4 and 2 replicates by more than 5% in detected beta value.
(TIFF)

S8 Fig Screenshot from UCSC genome browser used to extract the positions of transcription start sites. (TIFF)

S9 Fig Comparison in estimated 5hmC between oxBS and TAB arrays [3]. (TIFF)

S10 Fig Comparison in estimated 5hmC between the current study and the 5hmC reported in Stewart et al. 2014 [7]. (TIFF)

S1 Table Sample sheet for array design. (PDF)

S2 Table 5hmC percentages detected by qPCR and 450k array. (PDF)

S3 Table Details of primers used for qPCR validation. (PDF)
