# Supplementary material for: Accurate Measurement of 5-Methylcytosine and 5-Hydroxymethylcytosine in Human Cerebellum DNA by Oxidative Bisulfite on an Array (OxBS-Array)
Source: PLoS One. 2015 Feb 23;10(2):e0118202. doi: 10.1371/journal.pone.0118202 (PMC4338296; doi:10.1371/journal.pone.0118202)
Supplement: S1 Document — (PDF) [file pone.0118202.s001.pdf]

Accurate measurement of 5-methylcytosine and 5-hydroxymethylcytosine in human cerebellum DNA by oxidative bisulfite on an array (OxBS-array)

S. Field, D. Beraldi, M. Bachman, S. K. Stewart, S. Beck, S. Balasubramanian

November 12, 2014

Contents

1 Array analysis **1**
1.1 Importing and quality control . . . 1
1.2 Array clustering and library reproducibility . . . 2
1.3 Annotate probes . . . 3
1.4 Identification of 5hmC . . . 4
1.5 5hmC in CpG islands, shores and shelves . . . 6
1.6 Effect of replicates . . . 9
1.7 5hmC by Gene and Gene Ontology analysis . . . 11
1.8 GO Analysis . . . 12
1.9 Comparison oxBS vs TAB arrays . . . 13
1.10 Comparison to brain sample in Stewart *et al.* . . . 14
1.11 Validation oligonucleotides for qPCR assays . . . 14
1.12 Data submission to GEO . . . 15

2 References **17**

3 Supplementary Figures **18**

4 Appendix **23**
4.1 Additional functions . . . 23
4.2 qPCR primers . . . 25

The LaTeX code of this document can be accessed at `http://github.com/dariober/Illumina-450K-oxBS`

1 Array analysis

1.1 Importing and quality control

The starting point for this analysis are the raw `idat` files produced by the Illumina platform along with the corresponding sample sheet (Table S1). In the following pipeline `idat`, files and sample sheet are assumed to be in directory `../450k_array_data`. All output is produced in the current working directory. The Bioconductor package `minfi` [2] is assumed to be already installed. Several other R and Bioconductor packages are required along with additional programs. All these are referenced as they become necessary and they are freely available. In terms of operating system, any Unix or Linux distribution should be suitable.

Table S1: Sample sheet for array design

	Sample_Name	Sample_Group	Basename
1	BS1	Bisulfite	9373551079_R01C01
2	BS2	Bisulfite	9373551079_R02C01
3	BS3	Bisulfite	9373551079_R03C01
4	BS4	Bisulfite	9373551079_R04C01
5	OX1	Oxidation Bisulfite	9373551079_R05C01
6	OX2	Oxidation Bisulfite	9373551079_R06C01
7	OX3	Oxidation Bisulfite	9373551079_R01C02
8	OX4	Oxidation Bisulfite	9373551079_R02C02

```
library(minfi)
baseDir<- '../450k_array_data/'

targets <- read.450k.sheet(baseDir)
targets$bs<- c('BS', 'BS', 'BS', 'BS', 'oxBS','oxBS', 'oxBS', 'oxBS')

RGset <- read.450k.exp(base = baseDir, targets = targets)
qcReport(RGset, sampNames= targets$Sample_Name,
sampGroups= targets$bs, pdf= 'qcReport.rgset.pdf')
```

After having loaded the data files and having produced an initial quality cantrol, we removed probes with detection pvalue <0.01 in any of the 8 arrays. We re-plot the beta density profiles after having removed low quality probes (Figure S1). Arrays were then normalized using the subset within array normalization (SWAN [5])

```
detP<- detectionP(RGset)
failed<- detP > 0.01

## Keep probes which failed in at most this many arrays (0 = the probe passed in all arrays)
maxFail<- 0
mset <- preprocessRaw(RGset)
mset<- mset[rowSums(failed) <= maxFail, ]
MSet.swan<- preprocessSWAN(RGset, mSet = mset)

pdf('densityPlot.msetSwan.pdf')
densityPlot(MSet.swan, sampGroups= targets$bs,
main= sprintf('Beta values for filtered probes (n= %s)', nrow(MSet.swan)))
dev.off()

# save.image('MSet.swan.20140113.Rdata')
```

1.2 Array clustering and library reproducibility

To visually inspect the overall consistency between arrays and identify unusal samples, we applied principal components analysis (Figure S2) and hierarchical clustering (Figure S3) to the methylation levels. As shown in Figure S3 and Figure S2, no clear outliers were detected.

```
# load('MSet.swan.20140113.Rdata')

b<- getBeta(MSet.swan)

pcaResult<-prcomp(t(b))

pdf('pca.plot.pdf', w= 14/2.54, h= 14/2.54)
par(las= 1, mgp= c(2, 0.7, 0))
plot(pcaResult$x,
    main= 'Principal components from most variable beta values',
```

```
    xlab= sprintf('PC1 (sd: %s%%)',
     round(100 * (pcaResult$sdev[1] / sum(pcaResult$sdev)))),
    ylab= sprintf('PC2 (sd: %s%%)',
     round(100 * (pcaResult$sdev[2] / sum(pcaResult$sdev)))),
    pch= 19, col= ifelse(targets$bs=="BS", "blue", "red")
)
grid(col= 'grey30')
text(x= pcaResult$x[, "PC1"] + 4,
y= pcaResult$x[, "PC2"],
labels= targets$Sample_Name, cex= 1)
dev.off()

pdf('beta_dendrogram.pdf')
plot(hclust(dist(t(b))), labels= targets$Sample_Name)
dev.off()

# save.image('MSet.swan.20140113.Rdata')
```

We also assessed library reproducibility by pairwise correlation of beta values (see Figure 1 in main text).

```
# load('MSet.swan.20140113.Rdata')
library(corrplot)
corpct<- round(as.matrix(cor(getBeta(MSet.swan))) * 100, 1)
colnames(corpct)<- targets$Sample_Name
rownames(corpct)<- targets$Sample_Name

diag(corpct)<- min(corpct)

col<- colorRampPalette(c("yellow", "yellow", "orange", "red"))

pdf('corrplot_beta.pdf', pointsize= 12)
par(xpd= NA)
corrplot(corpct, type= "upper", order="alphabet", cl.lim= c(min(corpct),
max(corpct)), diag= FALSE,
    col=col(20), addCoefasPercent = FALSE,
    addCoef.col="black", tl.col="black", is.corr= FALSE,
    method= 'circle', mar= c(0,0,4,0),
    main= 'Pairwise correlation of Beta values')
dev.off()
# save.image('MSet.swan.20140113.Rdata')
```

1.3 Annotate probes

We annotated probes by assigning them to the nearest genomic feature. After retrieving probe positions, we used `annovar` [8] to link probe positions to genes and genomic features.

```
# load('MSet.swan.20140113.Rdata')
mapped <- mapToGenome(MSet.swan)
probPos<- as.data.frame(mapped@rowData)
probPos$probe_id<- rownames(probPos)
# save.image('MSet.swan.20140113.Rdata')
```

Format table for annovar and annotate:

```
# load('MSet.swan.20140113.Rdata')
probPos$ref<- 0
probPos$obs<- 0
annovar<- probPos[, c('seqnames', 'start', 'end', 'ref', 'obs', 'probe_id')]
write.table(file= '450K_probe_pos.bed', annovar, col.names= FALSE,
row.names= FALSE, quote= FALSE, sep= '\t')
save.image('MSet.swan.20140113.Rdata')
quit(save= 'no')
```

```
## Annotate probes
annotate_variation.pl --precedence upstream,utr5 --geneanno \
--buildver hg19 -dbtype gene 450K_probe_pos.bed [/path/to]/humandb
```

In addition, we assigned to each probe the nearest CpG island. The positions of CpG islands for human reference sequence hg19 were downloaded from the UCSC genome browser (table `cpgIslandExt.bed` saved locally as `hg19.cpgIslandExt.bed`). The program `closestBed` is part of the `bedtools` suite [6].

```
awk 'BEGIN{OFS="\t"} {print $3, $4-1, $5, $8, $1, $2}' 450K_probe_pos.bed.variant_function \
| closestBed -t first -a - -b ~/reference_seqs/hg19.cpgIslandExt.bed -D ref \
| gzip > 450K_probe_pos.annovar.bed.gz
```

1.4 Identification of 5hmC

To identify and quantify hydroxymethylation we tested the difference in M-values (logit of the beta values) between BS arrays and oxBS arrays (Figure S5). The function `aggSamp` is defined in section 4.1.

```
# load('MSet.swan.20140113.Rdata')
library(minfi)
M<- getM(MSet.swan, type= "beta", betaThreshold = 0.001)

x<- which(targets$bs == 'BS' | targets$bs == 'oxBS')

dmp_BS<- dmpFinder(M[,x], pheno= targets$bs[x], type="categorical",
shrinkVar= TRUE)

bvalues<- aggSamp(X= getBeta(MSet.swan)[,x], sampGroup= targets$bs[x])
dmp_BS<- cbind(dmp_BS, bvalues[rownames(dmp_BS),])
dmp_BS$hmc<- dmp_BS$BS - dmp_BS$oxBS
dmp_BS$probe_id<- row.names(dmp_BS)

dmp_BS$chrom<- probPos$seqnames[match(dmp_BS$probe_id, probPos$probe_id)]
dmp_BS$start<- probPos$start[match(dmp_BS$probe_id, probPos$probe_id)]

write.table(dmp_BS, gzfile('bs_vs_oxbs.dmpFinder.txt.gz'),
    row.names= FALSE, col.names= TRUE, sep= '\t', quote= FALSE)

## Code for Volcano plots
## ----------------------
pdf('volcano_bs_vs_oxbs.pdf', w= 16/2.54, h= 8/2.54, pointsize= 9)
par(mfrow= c(1,2), las= 1, mgp= c(2, 0.7, 0), mar= c(4, 3, 2, 0.5))
fdr<- 1
filter<- which(dmp_BS$qval <= fdr)
smoothScatter(x= 100 * dmp_BS$hmc[filter],
    y= -log10(dmp_BS$qval[filter]),
    colramp= colorRampPalette(
c("white", "lightblue", "red", "orange", "yellow")),
xlab= '% (BS - oxBS)',
ylab= '-log10(q-value)',
main= sprintf('Probes with fdr < %s n= %s', fdr, length(filter))
)
grid(col= 'grey60')

fdr<- 0.01
filter<- which(dmp_BS$qval <= fdr)
smoothScatter(x= 100 * dmp_BS$hmc[filter],
    y= -log10(dmp_BS$qval[filter]),
    colramp= colorRampPalette(
c("white", "lightblue", "red", "orange", "yellow")),
xlab= '% (BS - oxBS)',
ylab= '-log10(q-value)',
main= sprintf('Probes with fdr < %s n= %s', fdr, length(filter))
)
grid(col= 'grey60')
```

```
dev.off()

# save.image('MSet.swan.20140113.Rdata')
```

Note that without variance shrinkage, i.e. by setting `dmpFinder(...shrinkVar= FALSE)` the number of probes with significant differential methylation is reduced to 80803 .

```
x<- which(targets$Sample_Name %in% c('BS1', 'BS3', 'OX1', 'OX3'))
dmp_s13<- dmpFinder(M[,x], pheno= targets$bs[x], type="categorical",
shrinkVar= FALSE)
sum(dmp_s13$qval < 0.05)

x<- which(targets$Sample_Name %in% c('BS2', 'BS4', 'OX2', 'OX4'))
dmp_s24<- dmpFinder(M[,x], pheno= targets$bs[x], type="categorical",
shrinkVar= FALSE)
sum(dmp_s24$qval < 0.05)
```

By plotting the p-values for the difference BS − oxBS, we can see that there is no evidence of a negative differences, as expected under the null hypothesis that negative differences arise by chance (Figure S4).

```
pdf('histPosNeg.bs_vs_oxbs.pdf')
histPosNeg(dmp_BS$BS - dmp_BS$oxBS, pval= dmp_BS$pval)
dev.off()
```

Figure 4 in main text was produced with the following code:

```
ann<- read.table('450K_probe_pos.annovar.bed.gz', stringsAsFactors= FALSE, sep= "\t",
    col.names= c("chrom", "start", "end", "probe_id", "region", "gene_name",
        "chrom.cpg", "start.cpg", "end.cpg", "name.cpg", "dist.cpg"))
dmpann<- merge(dmp_BS, ann, by= "probe_id", sort= FALSE)

regions<- c('downstream', 'exonic', 'intergenic', 'intronic', 'upstream', 'UTR3', 'UTR5')
ncRna<- c('ncRNA_exonic', 'ncRNA_intronic', 'ncRNA_splicing', 'ncRNA_UTR3', 'ncRNA_UTR5')
other<- c('splicing', 'exonic;splicing', 'UTR5;UTR3', 'upstream;downstream')

dmpann$region<- ifelse(dmpann$region %in% ncRna, "ncRNA", dmpann$region)
dmpann$region<- ifelse(dmpann$region %in% other, "Other", dmpann$region)

regions_all<- aggregate(dmpann$probe_id, list(dmpann$region), length)
names(regions_all)<- c("region", "cnt.all")
filter<- which(dmpann$hmc > 0 & dmpann$qval < 0.01)
regions_hmc<- aggregate(dmpann$probe_id[filter], list(dmpann$region[filter]), length)
names(regions_hmc)<- c("region", "cnt.hmc")
regions_tab<- merge(regions_all, regions_hmc)
regions_tab$pct.hmc.region<- 100 * (regions_tab$cnt.hmc / regions_tab$cnt.all)
regions_tab$pct.hmc<- 100 * (regions_tab$cnt.hmc / sum(regions_tab$cnt.hmc))
regions_tab<- regions_tab[order(regions_tab$cnt.all, decreasing= TRUE),]

pdf("barplot_hmc_region.pdf", w= 12/2.54, h= 18/2.54, pointsize= 10)
par(mfrow= c(2,1), las= 1, mar= c(1,1,1,1), oma= c(6,4,2,1), mgp= c(2, 0.7, 0))
barplot(regions_tab$cnt.all, col= "#FF000050", border= NA)
grid(col= "grey30", nx= NA, ny= NULL)
mtext(text= "Number of probes", side= 2, las= 3, line= 3.5)
b<- barplot(regions_tab$pct.hmc.region, col= "#0000FF50", border= NA)
grid(col= "grey30", nx= NA, ny= NULL)
mtext(text= "% 5hmC probes", side= 2, las= 3, line= 3.5)
text(x= b, y= -2, labels= regions_tab$region, srt= 45, xpd= NA, adj= c(1, 0))
dev.off()
```

Figure 5 in main text was produced with the following code:

```
ann<- read.table('450K_probe_pos.annovar.bed.gz',
```

```
   col.names= c("chrom", "start", "end", "probe_id", "region", "gene_name",
   "cpgi_chrom", "cpgi_start", "cpgi_end", "cpgi_name", "cpgi_dist"),
   stringsAsFactors= FALSE, sep= '\t')

dmpann<- merge(dmp_BS, ann, by= c("probe_id"), sort= FALSE)

hmc<- dmpann[dmpann$qval < 0.01 & dmpann$hmc > 0, ]

## Put small categories in "Other" group
regions<- c('downstream', 'exonic', 'intergenic', 'intronic', 'upstream', 'UTR3', 'UTR5')
ncRna<- c('ncRNA_exonic', 'ncRNA_intronic', 'ncRNA_splicing', 'ncRNA_UTR3', 'ncRNA_UTR5')
other<- c('splicing', 'exonic;splicing', 'UTR5;UTR3', 'upstream;downstream')
hmc$region<- ifelse(hmc$region %in% ncRna, "ncRNA", hmc$region)
hmc$region<- ifelse(hmc$region %in% other, "Other", hmc$region)

medhmc<- aggregate(hmc$hmc, list(hmc$region), median)
names(medhmc)<- c("region", "median")
medhmc$count<- aggregate(hmc$hmc, list(hmc$region), length)$x

## This is to make boxplot() order boxes by median
hmc$region<- factor(hmc$region, levels= medhmc$region[order(medhmc$median)])

pdf('boxplot_hmc_regions.pdf', w= 16/2.54, h=12/2.54, pointsize= 9)
par(mar= c(5, 4, 1, 0.5), las= 2, mgp= c(2.5, 1, 0), mfrow= c(1, 1), xaxt= 'n', xpd= NA)
boxplot(hmc$hmc*100 ~ hmc$region, pch= '.', ylab= "% 5(h)mC", col= 'pink',
    border= "grey1", varwidth= FALSE, at= seq(1, nrow(medhmc))-0.3, boxwex= 0.3,
    ylim= c(0, max(hmc$oxBS*100)), names= NA)
text(srt= 45, x= 1:nrow(medhmc), y= -7, labels= medhmc$region[order(medhmc$median)],
    adj= c(1,0))
par(xpd= FALSE)
grid(ny= NULL, nx= NA, col= 'grey10')

boxplot(hmc$oxBS*100 ~ hmc$region, pch= '.', ylab= "", col= 'lightblue',
    border= "grey1", varwidth= FALSE, add= TRUE, at= seq(1, nrow(medhmc))+0,
    boxwex= 0.3, names= NA)
grid(ny= NULL, nx= NA, col= 'grey10')
dev.off()
```

For the definition of regions in Figures 4 and 5, see the description of output files from annovar (`http://www.openbioinformatics.org/annovar/annovar_gene.html`)

Figure 7 in main text was produced with the code:

```
Lab.palette <- colorRampPalette(c("white", "lightblue", "orange", "red"), space = "Lab")
x<- dmp_BS$oxBS[dmp_BS$qval < 0.01 & dmp_BS$hmc > 0]*100
y<- dmp_BS$hmc[dmp_BS$qval < 0.01 & dmp_BS$hmc > 0]*100
pdf("scatter_hmc_vs_mc_all.pdf", w= 8/2.54, h= 8/2.54, pointsize= 9)
par(las=1, mgp= c(2, 0.6, 0), mar= c(3, 3, 1.5, 0.5), cex.main= 1)
    smoothScatter(x, y, colramp = Lab.palette, nrpoints= 200,
        xlab= '5mC', ylab= '5hmC', main= sprintf("5hmC probes n= %s", length(y)))
    lines(loess.smooth(x, y, span= 0.1), col= 'dodgerblue', lwd= 2)
dev.off()
```

1.5 5hmC in CpG islands, shores and shelves

Shores were defined as the regions extending for 2 kb from the border of CpG islands. Similarly, shelves extend shores by 2 kb. Positions of CpG islands and chromosome sizes were downloaded from UCSC genome browser. The program `slopBed` is part of the `bedtools` suite [6].

```
sed 's/: /_/' [/path/to]/hg19.cpgIslandExt.bed \
| awk 'BEGIN{FS="\t"; OFS="\t"}
    {print $1, $2-2000, $2, $4"_shore_up"}
    {print $1, $2-4000, $2-2000, $4"_shelf_up"}
    {print $1, $3, $3+2000, $4"_shore_down"}
    {print $1, $3+2000, $3+4000, $4"_shelf_down"}
    {print $0} ' \
```

```
| awk 'BEGIN{FS="\t"; OFS="\t"}$3 > 0 {if($2 < 0) print $1, 0, $3, $4; else print $0}' \
| slopBed -b 0 -g [/path/to]/hg19.chromInfo.txt -i - \
| awk 'BEGIN{FS="\t"; OFS="\t"}$3 > $2 {if($2 < 0) print $1, 0, $3, $4; else print $0}' \
| sort -k1,1 -k2,2n -k3,3n > hg19.cpgIslandExtShoresShelves.bed
```

Produce boxplots in CpG islands, shelves and shores as reported in Figure 6 (main text):

```
# load('MSet.swan.20140113.Rdata')
cpg<- read.table('hg19.cpgIslandExtShoresShelves.bed', header= FALSE,
    stringsAsFactors= FALSE, sep= "\t", col.names= c("chrom", "start", "end", "name"))

dmpbed<- data.frame(chrom= dmp_BS$chrom, start= dmp_BS$start-1, end= dmp_BS$start,
    probe_id= dmp_BS$probe_id)
dmpcpg<- bedtools.intersect(dmpbed, cpg, "-wa -wb")
names(dmpcpg)<- c("chrom", "start", "end", "probe_id", "chrom.cpg", "start.cpg",
    "end.cpg", "cpg")
dmpcpg<- merge(dmpcpg, dmp_BS, by.x= c("probe_id"), by.y= c("probe_id"))
dmpcpg$cpg_feature<- sub("CpG_\\d+_", "", dmpcpg$cpg, perl= TRUE)
dmpcpg$cpg_feature<- sub("CpG_\\d+", "CpGi", dmpcpg$cpg, perl= TRUE)

## Assign probes to one feature only given the precedence vector:
library(data.table)
precedence<- c('CpGi', 'CpGi_shore_down', 'CpGi_shore_up', 'CpGi_shelf_down', 'CpGi_shelf_up')
dmpcpg<- data.table(dmpcpg)
setkeyv(dmpcpg, "cpg_feature")

oo<- data.table(rank= 1:length(precedence), precedence)
setkey(oo, "precedence")

## Add rank to each level of precedence
dmpcpg2<- dmpcpg[oo]
dmpcpgRank<- dmpcpg2[, list(rank= min(rank)), by= probe_id]
DTdedup<- merge(dmpcpg2, dmpcpgRank, by= c("probe_id", "rank"))

## In DTdedup you still have the same probe mapped to different CpG features.
## E.g. DTdedup[probe_id == "cg00002769", ]
## Arbitrarily assign to each probe the feature with lower coords.
dmpcpgPos<- dmpcpg2[, list(start.cpg= min(start.cpg)), by= probe_id]
DTdedup<- merge(DTdedup, dmpcpgPos, by= c("probe_id", "start.cpg"))

stopifnot(length(DTdedup$probe_id) == length(unique(DTdedup$probe_id)))

dmpcpg<- DTdedup

## Give names which will be in the right order on boxplot
cpg_feature2<- ifelse(dmpcpg$cpg_feature == "CpGi_shore_up", "B CpGi_shore_up",
ifelse(dmpcpg$cpg_feature == "CpGi_shore_down", "D CpGi_shore_down",
ifelse(dmpcpg$cpg_feature == "CpGi", "C CpGi",
ifelse(dmpcpg$cpg_feature ==  "CpGi_shelf_down", "E CpGi_shelf_down",
ifelse(dmpcpg$cpg_feature ==  "CpGi_shelf_up", "A CpGi_shelf_up", NA)))))

cpgnames<- sub('.* ', '', sort(unique(cpg_feature2)), perl= TRUE)

filter<- rep(TRUE, nrow(dmpcpg)) # dmpcpg$qval < 0.01 & dmpcpg$hmc > 0
pdf("boxplot_cpgShoresShelves.pdf", w= 16/2.54,  h= 10/2.54, pointsize= 9)
par(las= 2, xpd= NA, mar= c(8, 4, 2, 0.5), mgp= c(2.5, 1, 0), mfrow= c(1, 2), cex.main= 1)
b<- boxplot(100 * dmpcpg$hmc[filter] ~ cpg_feature2[filter], names= NA,
    ylim= c(100 * min(dmpcpg$hmc[filter]), 100 * max(dmpcpg$hmc[filter])),
    ylab= '% 5hmC', col= 'pink', border= 'grey20', pch= '.',
    main= sprintf('All 5hmC (n= %s)', length(dmpcpg$hmc[filter])), varwidth= TRUE)
text(x= 1:length(cpgnames), y= par('usr')[3] - 3, labels= cpgnames, srt= 45, adj= c(1,0))

b<- boxplot(100 * dmpcpg$oxBS ~ cpg_feature2, names= NA,
    ylim= c(0, 100 * max(dmpcpg$oxBS)), ylab= '% 5mC', col= 'lightblue', border= 'grey20', pch= '.',
    main= sprintf('All 5mC (n= %s)', length(dmpcpg$oxBS)), varwidth= TRUE)
text(x= 1:length(cpgnames), y= -8, labels= cpgnames, srt= 45, adj= c(1,0))
dev.off()

## Probes in CpGi, shores and shelves. significant and non sig
inCpG<- nrow(dmpcpg) / nrow(dmp_BS)
inCpGSig<- nrow(dmpcpg[dmpcpg$qval < 0.01 & dmpcpg$hmc > 0,])
inCpGSig / nrow(dmpcpg)
outCpGSig<- nrow(dmp_BS[dmp_BS$qval < 0.01 & dmp_BS$hmc > 0,])
```

```
# save.image('MSet.swan.20140113.Rdata')
```

Similar to CpG islands, we profiled 5(h)mC in and around transcription start sites (TSS, Figure 6 in main text). The TSS positions have been extracted from UCSC Genome Browser (see also FigureS8). Profile matrices were computed with the deepTools suite as shown below (`https://github.com/fidelram/deepTools`)

```
zcat hg19.refGene.tss.txt.gz \
| awk 'BEGIN{OFS="\t"} NR > 1 {if($3 =="+") {start=$4; end=$4+1}
    else {start=$4-1; end=$4} print $2, start, end, ".", ".", $3}' \
| sort -k1,1 -k2,2n -k3,3n \
| uniq > hg19.refGene.tss.bed

## bigWig for 5hmC
zcat bs_vs_oxbs.dmpFinder.txt.gz \
| awk 'BEGIN{OFS= "\t"} NR > 1 {print $11, $12-1, $12, $8 * 100}' \
| sort -k1,1 -k2,2n -k3,3n > /tmp/bs_vs_oxbs.dmpFinder.hmc.bedGraph &&
bedGraphToBigWig /tmp/bs_vs_oxbs.dmpFinder.hmc.bedGraph \
    ~/reference_seqs/hg19.chromInfo.txt bs_vs_oxbs.dmpFinder.hmc.bw &&
rm /tmp/bs_vs_oxbs.dmpFinder.hmc.bedGraph

## bigWig for 5mC
zcat bs_vs_oxbs.dmpFinder.txt.gz \
| awk 'BEGIN{OFS= "\t"} NR > 1 {print $11, $12-1, $12, $7 * 100}' \
| sort -k1,1 -k2,2n -k3,3n > /tmp/bs_vs_oxbs.dmpFinder.mc.bedGraph &&
bedGraphToBigWig /tmp/bs_vs_oxbs.dmpFinder.mc.bedGraph \
    ~/reference_seqs/hg19.chromInfo.txt bs_vs_oxbs.dmpFinder.mc.bw &&
rm /tmp/bs_vs_oxbs.dmpFinder.mc.bedGraph

## 5hmC
bsub -R "rusage[mem=4096] hosts[span=1]" -n 8 -oo deeptools.hmc.log "computeMatrix reference-point \
    -p 8 \
    -R hg19.refGene.tss.bed \
    -S bs_vs_oxbs.dmpFinder.hmc.bw \
    -out bs_vs_oxbs.dmpFinder.hmc.out.gz \
    -bs 50 \
    -b 5000 \
    -a 5000"

## 5mC
bsub -R "rusage[mem=4096] hosts[span=1]" -n 8 -oo deeptools.mc.log "computeMatrix reference-point \
    -p 8 \
    -R hg19.refGene.tss.bed \
    -S bs_vs_oxbs.dmpFinder.mc.bw \
    -out bs_vs_oxbs.dmpFinder.mc.out.gz \
    -bs 50 \
    -b 5000 \
    -a 5000"

## Plot profile
profiler -m bs_vs_oxbs.dmpFinder.hmc.out.gz \
    -out tss.hmc.profile.pdf \
    --outFileNameData tss.hmc.profile.tab \
    --plotHeight 8 \
    --plotWidth 16 \
    --startLabel 'up-TSS' \
    --endLabel 'down-TSS' \
    --plotType lines \
    --yMin 0 \
    --regionsLabel '' \
    --yAxisLabel '% 5hmC'

profiler -m bs_vs_oxbs.dmpFinder.mc.out.gz \
    -out tss.mc.profile.pdf \
    --outFileNameData tss.mc.profile.tab \
    --plotHeight 8 \
    --plotWidth 16 \
    --startLabel 'up-TSS' \
    --endLabel 'down-TSS' \
    --plotType lines \
    --yMin 0 \
```

```
    --regionsLabel '' \
    --yAxisLabel '% 5mC'

## Re-plot profile
R
mc<- read.table('tss.mc.profile.tab', skip= 1, col.names= c("bin", "avg", "sd"))
hmc<- read.table('tss.hmc.profile.tab', skip= 1, col.names= c("bin", "avg", "sd"))
tab<- merge(mc, hmc, by= "bin", suffixes= c(".mc", ".hmc"))

pdf('tss.profile.pdf', w= 16/2.54, h= 11/2.54)
par(las= 1, mgp= c(2, 0.7, 0), mar= c(4, 4, 1, 0.5))
    plot(tab$bin, tab$avg.mc, type= 'l', ylim= c(0, max(tab$avg.mc)), lwd= 2,
        col= 'blue', ylab= "% 5(h)mC", xlab= "TSS position")
    points(tab$bin, tab$avg.hmc, type= 'l', lwd= 2, col= 'red')
    grid(col= "grey30")
    legend('bottomleft', legend= c("5mC", "5hmC"), col= c('blue', 'red'), lwd= 2,
        bty= "n", cex= 0.85)
dev.off()
```

1.6 Effect of replicates

We investigated the impact of using 2 instead of 4 replicates on the detection and quantification of 5hmC (Figure 2 in main text).

```
library(VennDiagram)
library(minfi)

# load('MSet.swan.20140113.Rdata')
M<- getM(MSet.swan, type= "beta", betaThreshold = 0.001)

subsets<- list(
    "dmp_full"= 1:nrow(targets),
    "dmp_s1"= which(targets$Sample_Name %in% c('BS1', 'BS3', 'OX1', 'OX3')),
    "dmp_s2"= which(targets$Sample_Name %in% c('BS2', 'BS4', 'OX2', 'OX4'))
)

dmp_subsets<- list()
for(i in 1:length(subsets)){
    sname<- names(subsets)[i]
    print(sname)
    x<- unlist(subsets[i])
    dmp<- dmpFinder(M[, x], pheno= targets$bs[x], type="categorical", shrinkVar= TRUE)
    bvalues<- aggSamp(X= getBeta(MSet.swan)[,x], sampGroup= targets$bs[x])
    dmp<- cbind(dmp, bvalues[rownames(dmp),])
    dmp$hmc<- dmp$BS - dmp$oxBS
    dmp$probe_id<- row.names(dmp)
    dmp_subsets[[sname]]<- dmp
}
dmp_full<- dmp_subsets[["dmp_full"]]
dmp_s1<- dmp_subsets[["dmp_s1"]]
dmp_s2<- dmp_subsets[["dmp_s2"]]

## Detection limits in full set and subsets
quantile(dmp_full$hmc[dmp_full$hmc > 0 & dmp_full$qval < 0.01] * 100)
quantile(dmp_s1$hmc[dmp_s1$hmc > 0 & dmp_s1$qval < 0.01] * 100)
quantile(dmp_s2$hmc[dmp_s2$hmc > 0 & dmp_s2$qval < 0.01] * 100)

pthr<- 0.01
af<- sum(dmp_full$qval < pthr) ##Sign in 1,2,3,4
a1<- sum(dmp_s1$qval < pthr) ## Sign in 1,3
a2<- sum(dmp_s2$qval < pthr) ## Sign in 2,4

nf1<- sum(rownames(dmp_full)[dmp_full$qval < pthr]
%in% rownames(dmp_s1)[dmp_s1$qval < pthr])
nf2<- sum(rownames(dmp_full)[dmp_full$qval < pthr]
%in% rownames(dmp_s2)[dmp_s2$qval < pthr])

n12<- sum(rownames(dmp_s1)[dmp_s1$qval < pthr] %in%
rownames(dmp_s2)[dmp_s2$qval < pthr])
```

```
nf12<- length(Reduce(intersect, list(
    rownames(dmp_full)[dmp_full$qval < pthr],
    rownames(dmp_s1)[dmp_s1$qval < pthr],
    rownames(dmp_s2)[dmp_s2$qval < pthr])
))

pdf('venn.4reps_vs_2reps_A.pdf', w= 6/2.54,  h= 6/2.54, pointsize= 9)
venn.plot<- draw.pairwise.venn(
    af, a1, nf1,
    c("Reps 1,2,3,4", "Reps 1,3"), col= c("transparent", "transparent"),
    fill= c("blue", "green"), alpha= 0.30,
    fontfamily= 'sans', cat.fontfamily = "sans", fontface= 1, cat.fontface = 1,
    margin= c(0.05,0.05,0.05,0.05)
)
grid.draw(venn.plot);
dev.off()

pdf('venn.4reps_vs_2reps_B.pdf', w= 6/2.54,  h= 6/2.54, pointsize= 9)
venn.plot<- draw.pairwise.venn(
    af, a2, nf2,
    c("Reps 1,2,3,4", "Reps 2,4"), col= c("transparent", "transparent"),
    fill= c("blue", "red"), alpha= 0.30,
    fontfamily= 'sans', cat.fontfamily = "sans", fontface= 1, cat.fontface = 1,
    margin= c(0.05,0.05,0.05,0.05)
)
grid.draw(venn.plot);
dev.off()

pdf('venn.2reps_A_vs_2reps_B.pdf', w= 6/2.54,  h= 6/2.54, pointsize= 9)
venn.plot<- draw.pairwise.venn(
    a1, a2, n12,
    c("Reps 1,3", "Reps 2,4"), col= c("transparent", "transparent"),
    fill= c("green", "red"), alpha= 0.30,
    fontfamily= 'sans', cat.fontfamily = "sans", fontface= 1, cat.fontface = 1,
    margin= c(0.05,0.05,0.05,0.05)
)
grid.draw(venn.plot);
dev.off()

# save.image('MSet.swan.20140113.Rdata')
```

We can see how two replicates reduced the sensistivity of the analysis in detecting 5hmC (Figure S6):

```
pdf('density_subsets.pdf', w= 12/2.54, h= 12/2.54, pointsize= 9)
par(mgp= c(2.5, 0.7, 0), las= 1)
    filter<- which(dmp_full$qval < 0.01)
    plot(density(100 * dmp_full$hmc[filter]), col= 'red', lwd= 2, ylim= c(0, 0.12),
        xlab= '% (BS - oxBS)', main= 'Effect of signal detected')
    print( median(100 * dmp_full$hmc[filter]) ) # 14.0327
    filter<- which(dmp_s1$qval < 0.01)
    lines(density(100 * dmp_s1$hmc[filter]), col= 'blue', lwd= 2)
    print( median(100 * dmp_s1$hmc[filter]) )   # 19.80764
    filter<- which(dmp_s2$qval < 0.01)
    lines(density(100 * dmp_s2$hmc[filter]), col= 'grey30', lwd= 2)
    print( median(100 * dmp_s2$hmc[filter]) )   # 19.38871
    grid(col= 'grey30')
    legend('topright', legend= c('4 reps', 'reps 1 & 3', 'reps 2 & 4'),
        col= c('red', 'blue', 'grey30'), lwd= 2)
dev.off()

full_probes<- dmp_full$probe_id[dmp_full$qval < 0.01]
s1_probes<- dmp_s1$probe_id[dmp_s1$qval < 0.01]
s1full<- intersect(full_probes, s1_probes)

hmcFull<- 100 * dmp_full$hmc[dmp_full$probe_id %in% s1full]
hmcS1<- 100 * dmp_s1$hmc[dmp_s1$probe_id %in% s1full]

pdf('scatter_full_vs_s1.pdf', w= 14/2.54, h= 14/2.54, pointsize= 10)
par(las= 1, mgp= c(2.5, 0.7, 0), mar= c(4, 6, 4, 0.1))
smoothScatter(hmcFull, hmcS1, xlab= '4 replicates\n[BS - oxBS]',
    ylab= '2 replicates\n[BS - oxBS]',
    colramp= colorRampPalette(c("white", "lightblue", "red", "orange", "yellow")),
    main= 'Difference between BS and oxBS using 4 or 2 replicates', nrpoints= 1000)
```

```
lines(loess.smooth(hmcFull, hmcS1, span= 0.05), lwd= 2, col= 'blue')
polygon(c(-100, 100, 100, -100), y = c(-105, 95, 105, -95),
    col= makeTransparent("grey", 0.4), border= NA)
grid(col= 'grey30')
dev.off()
```

1.7 5hmC by Gene and Gene Ontology analysis

We performed gene ontology (GO) analysis by summarizing 5-(hydroxy)methylation within genes and testing for overrepresentation of GO terms.

```
library(data.table)

# load('MSet.swan.20140113.Rdata')
ann<- read.table('450K_probe_pos.annovar.bed.gz',
   col.names= c("chrom", "start", "end", "probe_id", "region", "gene_name",
   "cpgi_chrom", "cpgi_start", "cpgi_end", "cpgi_name", "cpgi_dist"),
   stringsAsFactors= FALSE, sep= '\t')

dmp<- merge(dmp_BS, ann, by= c("probe_id"), sort= FALSE)

## Include only probes in genes
regions<- c("upstream",  "UTR5", "exonic", "intronic", "UTR3")
gdmp<- dmp[dmp$region %in% regions, ]

gdmp$pval[gdmp$hmc < 0]<- 1 ## Reset to 1 pvalues for negative 5hmC
gdmp$qval[gdmp$hmc < 0]<- 1 ## Reset to 1 qvalues for negative 5hmC

## To combine pvalues use the average of log pvalue:
## NB: Negative hmC has pval= 1 as per above
combine.pval<- function(p){
    cp<- mean(log10(p))
    return(10^cp)
}

## To summarize 5hmC average 5hmC for significant probes only
gdmp$hmc.sig<- ifelse(gdmp$qval < 0.01 & gdmp$hmc > 0, gdmp$hmc, NA)

count.non.na<- function(x){
    ## Count non NA elements in vector x
    ## Use to count the number of significant hmC probes. NS probes are marked
    ## NA in vector x
    xlen<- sum(!is.na(x))
}
gdmp <- data.table(gdmp)

probesByGene<- gdmp[, list(cnt.probes= length(probe_id),
comb.p= combine.pval(pval),
cnt.hmc= count.non.na(hmc.sig),
pct.hmc= mean(hmc.sig, na.rm= TRUE),
pct.mc= mean(oxBS)),
by = c('gene_name')]

probesByGene<- as.data.frame(probesByGene)
probesByGene<- probesByGene[order(probesByGene$comb.p),]

write.table(probesByGene, 'probesByGene.txt', sep= '\t',
    col.names= TRUE, row.names= FALSE, quote= FALSE)

write.table(data.frame(
gdmp$chrom.x,
gdmp$start.x - 1,
gdmp$start.x,
gdmp$hmc*100, gdmp$gene_name),
    'hmc.dmp_genes.bedGraph', col.names= FALSE,
    row.names= FALSE, quote= FALSE, sep= '\t')

write.table(data.frame(gdmp$chrom.x,
gdmp$start.x - 1,
gdmp$start.x,
```

```
gdmp$oxBS*100,
gdmp$gene_name),
'mc.dmp_genes.bedGraph',
col.names= FALSE, row.names= FALSE, quote= FALSE, sep= '\t')

genesToPlot<- gdmp[, list(start= min(start.x)-1, end= max(start.x)),
    by = c('gene_name', 'chrom.x')]

## Consider genes with more than n probes
hmcGenes<- probesByGene$gene_name[probesByGene$cnt.hmc > 5][1:100]

genesToPlot<- genesToPlot[order(genesToPlot$chrom, genesToPlot$start)]
genesToPlot<- genesToPlot[gene_name %in% hmcGenes]
write.table(genesToPlot[, c('chrom.x', 'start', 'end', 'gene_name'), with= FALSE],
'genesToPlot.bed', sep= '\t', row.names= FALSE, col.names= FALSE, quote= FALSE)

probesByGene[probesByGene$cnt.hmc > 5, ][1:10,]


gdmp$region_id<- ifelse(gdmp$region == 'upstream', 'U',
    ifelse(gdmp$region == 'UTR5', '5',
    ifelse(gdmp$region == 'exonic', 'E',
    ifelse(gdmp$region == 'intronic', 'I',
    ifelse(gdmp$region == 'UTR3', '3', NA)))))

stopifnot(NA %in% unique(gdmp$region_id) == FALSE)
# save.image('MSet.swan.20140113.Rdata')
```

This code produces an overview of the level of 5hmC in the top hydroxymethylated ones as defined above.

```
load('MSet.swan.20140113.Rdata')
pdf('gene_hmc.pdf', w= 14/2.54, h= 8/2.54, pointsize= 9)
par(las= 1, mgp= c(2, 0.7, 0), bty= 'l')
for(gene in hmcGenes){
    print(gene)
    geneFilter<- which(gdmp$gene_name == gene)
    pdata<- gdmp[geneFilter,]
    pdata<- pdata[order(pdata$start.x),]
    plot(x= pdata$start.x, y= 100 * pdata$hmc, type= 'h',
        ylim= c(ifelse(min(pdata$hmc) > 0, 0, min(pdata$hmc)*100), max(pdata$hmc)*100 + 1),
        xlab= sprintf('%s (%s bp)', unique(pdata$chrom.x), diff(range(pdata$start.x))),
        ylab= '% hmC',
        main= unique(pdata$gene_name), col= 'blue', lwd= 2)
    points(x= pdata$start.x, y= 100 * pdata$hmc + 1, pch= '*',
     col= ifelse(pdata$qval < 0.01, 'red', NA))
    grid(nx= NA, ny= NULL, col= 'grey40')
    text(y= max(pdata$hmc)*100 + 5, x= pdata$start.x,
     labels= pdata$region_id, xpd= NA)
}
dev.off()
# save.image('MSet.swan.20140113.Rdata')
```

1.8 GO Analysis

We used DAVID [4] for detecting GO terms enriched in genes highly methylated or hydroxymethylated. The gene symbols assigned above have been first converted to entrez IDs

```
library(org.Hs.eg.db)
sym2eg<- org.Hs.egSYMBOL2EG
## Genes mapped to entrez
mapped_genes<- probesByGene$gene_name[probesByGene$gene_name %in% mappedkeys(sym2eg)]

symbol2id<- as.data.frame(sym2eg[mapped_genes])

## Note some genes have multiple entrez ids
which((table(symbol2id$symbol) > 1) == TRUE)
```

```
entrezProbes<- merge(symbol2id, probesByGene, by.x= 'symbol', by.y= 'gene_name',
    sort= FALSE)

## Criteria to establish top genes
entrezProbes<- entrezProbes[order(entrezProbes$comb.p), ]
entrezProbes<- entrezProbes[which(entrezProbes$cnt.probes > 5), ]
ntop<- 500

## Send to DAVID
## -------------
## BACKGROUND:
write.table(entrezProbes[, c('gene_id')], 'background.genes.txt', sep= '\t',
    row.names= FALSE, col.names= FALSE, quote= FALSE)

## HMC Top genes:
write.table(entrezProbes[1:ntop, c('gene_id')], 'top.hmc.genes.txt', sep= '\t',
    row.names= FALSE, col.names= FALSE, quote= FALSE)

## MC Top genes:
entrez_mc<- entrezProbes[order(entrezProbes$pct.mc, decreasing= TRUE), ]
write.table(entrez_mc[1:ntop, c('gene_id')], 'top.mc.genes.txt', sep= '\t',
    row.names= FALSE, col.names= FALSE, quote= FALSE)
# save.image('MSet.swan.20140113.Rdata')
```

Files `background.genes.txt` (background genes), `top.hmc.genes.txt` and `top.mc.genes.txt` (top 5hmC and 5mC genes, respectively) were manually uploaded to DAVID.

1.9 Comparison oxBS vs TAB arrays

Processed data from Chopra *et al.* 2014 [3] were downloaded from GEO, accession ID GSE49177, file `GSE49177_signal_intensities.txt.gz`. The correlation between oxBS and TAB method is in Figure S9.

```
load('MSet.swan.20140113.Rdata')
gse<- read.table('GSE49177_signal_intensities.txt.gz', sep= '\t', header= TRUE,
    stringsAsFactors= FALSE)

## Select Human brain arrays only
hs<- grep('E06.*', names(gse), perl= TRUE)
gse<- gse[, c(1, hs)]
rownames(gse)<- gse$ID_REF

gsepvals<- gse[, grep('.*\\.Pval$', names(gse), perl= TRUE)]
pvalFilter<- rowSums(gsepvals >=  0.01) ## Count how many arrays failed in each array
pvalFilter<- which(pvalFilter == 0)
gse<- gse[pvalFilter,]

## Check max of pval columns is < 0.01
apply(gse[, 2:ncol(gse)], 2, max)

## Calculate bvalaues
unmet<- gse[, grep('.*\\.Unmethylated\\.Signal$', names(gse), perl= TRUE)]
met<- gse[, grep('.*\\.Methylated\\.Signal$', names(gse), perl= TRUE)]

gseBeta<- met / (met + unmet + 100)
names(gseBeta)<- sub('.Methylated.Signal', '', names(gseBeta))

## Average hmC levels
tabhmc<- rowMeans(gseBeta[, grep('_TAB_', names(gseBeta))])

oxsig<- dmp_BS$probe_id[which(dmp_BS$qval < 0.01)]
tabOxbs<- merge(data.frame(probe_id= names(tabhmc), tab_hmc= tabhmc),
    dmp_BS[, c("probe_id", "hmc")], by.x= c("probe_id"), by.y= c("probe_id"))
tabOxbsSig<- tabOxbs[tabOxbs$probe_id %in% oxsig,]

Lab.palette <- colorRampPalette(c("white", "lightblue", "yellow", "orange", "red"),
    space = "Lab")
pdf("smooth_tab_vs_oxbs.pdf", w= 16/2.54, h= 16/2.54, pointsize= 9)
```

```
par(mgp= c(2.5, 0.7, 0), las= 1, cex= 1.2)
smoothScatter(tabOxbsSig$tab_hmc, tabOxbsSig$hmc, colramp = Lab.palette,
    nrpoints= 1000, xlim= c(0, 0.8),  ylim= c(-0.2, 0.8), xlab= 'TAB', ylab= 'oxBS',
    main= sprintf('n probes= %s', length(tabOxbsSig$tab_hmc)))
grid(col= 'grey60')
abline(a= 0, b= 1, col= 'red', lty= 'dotted')
lines(loess.smooth(tabOxbsSig$tab_hmc, tabOxbsSig$hmc, span= 0.01), col= 'blue', lwd= 2)
dev.off()

# save.image('MSet.swan.20140113.Rdata')
```

1.10 Comparison to brain sample in Stewart *et al.*

```
brain1<- read.table('AMSbioBrain_allMVPlist_2replicatesNewProtocol.txt', sep= '\t', header= TRUE)
comp<- merge(brain1, dmp_BS, by.x= 'probeID', by.y= 'probe_id')

cor(comp$deltaBeta, comp$hmc) ## 0.5851896
nrow(comp) ## 394197

colpal<- colorRampPalette(c("white", "lightblue", "yellow", "orange", "red"))
pdf('scatter_hmc_vs_stewart.pdf', w= 16/2.54, h= 16/2.54, pointsize= 9)
par(mgp= c(2.5, 0.7, 0), las= 1, cex= 1.2)
smoothScatter(comp$deltaBeta, comp$hmc, colramp= colpal, xlab= '5hmC [Stewart]',
    ylab= '5hmC [current]', main= '5hmC in brain detected in the current study vs\nthe dataset from Stewart et al.')
abline(a= 0, b= 1, col= 'grey30', lwd= 2, lty= 'dotted')
grid(col= 'grey30')
dev.off()
```

1.11 Validation oligonucleotides for qPCR assays

The oligonuclotides designed around the pool of probes used for validation have been tested for their uniqueness of sequence in the human genome. To this end, sequence similarity between oligonucleotides and any sequence in the genome has been tested via a blast [1] search as follows:

```
blastn -outfmt 6 \
    -db genome.fa \
    -query validation_oligos.20140215.fa \
    -out validation_oligos.20140215.blastn.txt \
    -word_size 7 \
    -evalue 100 \
    -task blastn
```

Where `validation_oligos.20140215.fa` is a fasta file of oligonucleotides to test and `genome.fa` is a fasta file for human reference sequence hg19 indexed for for blast. The complete list of primers with their details of sequence and position is in Table S3

```
trans.arcsine <- function(x){
  asin(sign(x) * sqrt(abs(x)))
}

qpcr<- read.table('450K_qPCR_comp.csv', header= TRUE)
qpcr<- qpcr[is.na(qpcr$pct.qPCR)==FALSE, ]
qpcr$probe_id<- sub("CG", "cg", qpcr$CpG)
qpcr<- merge(qpcr, dmp_BS[, c("probe_id", "hmc")])
qpcr<- qpcr[order(qpcr$pct.qPCR),]

## Correlation 450K - qPCR:
cor(qpcr$pct.450K, qpcr$pct.qPCR)

qpcr$pct.diff<- (trans.arcsine(qpcr$pct.450K) - trans.arcsine((qpcr$pct.qPCR + 0.0001))) /
    trans.arcsine((qpcr$pct.qPCR + 0.0001))
```

```
filter<- qpcr$pct.450K > 0

pdf("450K_qPCR_comp.pdf", w= 24/2.54, h= 13/2.54)
par(las= 1, mgp= c(2, 0.7, 0), mfrow= c(1,2))
plot(100 * qpcr$pct.qPCR, 100 * qpcr$hmc, pch= 19, col= "#FF00007F", cex= 1,
    xlab= "% 5hmC qPCR", ylab= "% 5hmC 450K")
abline(a= 0, b= 1, lty= "dotted", col= "blue", lwd= 1.5)
grid(col= "grey30")

plot(100 * qpcr$pct.qPCR[filter], 100 * qpcr$pct.diff[filter], pch= 19,
    col= "#FF00007F", cex= 1, xlab= "% 5hmC qPCR", ylab= "% Difference from qPCR")
abline(h= 0, lty= "dotted", col= "blue", lwd= 1.5)
grid(col= "grey30")
dev.off()
```

Table S2: 5hmC percentages detected by qPCR and 450k array

	probe_id	% qPCR	% 450k
1	cg02351555	0	-10
2	cg19841423	0	-10
3	cg22237200	0	-6
4	cg12641434	1	3
5	cg13529101	1	-12
6	cg16959747	8	10
7	cg18918390	8	10
8	cg10061770	9	10
9	cg19644590	9	10
10	cg12882907	10	10
11	cg02667291	18	20
12	cg12131862	19	20
13	cg27553486	19	20
14	cg13685679	20	20
15	cg08133755	21	20
16	cg09828580	27	30
17	cg26875137	27	30
18	cg06805880	28	30
19	cg01272627	29	30
20	cg07141452	30	30
21	cg09832245	30	30
22	cg10837846	30	30
23	cg18524262	30	40
24	cg14429457	34	36
25	cg08321942	35	36
26	cg22117062	36	38
27	cg16613029	37	37

In Figure 9 right panel (main text) the percentages 5hmC have been transformed to the square root of the arcsine as $\arcsin\left(sgn(x)\sqrt{|x|}\right)$ and negative percentages have been omitted.

1.12 Data submission to GEO

The data submitted to GEO (accession id GSE63179) has been produced in R as described here

```
library(minfi)
baseDir<- '../450k_array_data/'
targets <- read.450k.sheet(baseDir)
```

```
targets$bs<- c('BS', 'BS', 'BS', 'BS', 'oxBS','oxBS', 'oxBS', 'oxBS')
RGset <- read.450k.exp(base = baseDir, targets = targets)

detP<- detectionP(RGset)
colnames(detP)<- paste('array', colnames(detP), 'pval', sep= '.')
failed<- detP > 0.01
## Keep probes which failed in at most this many arrays (0 = the probe passed
## in all arrays)
maxFail<- 0
mset <- preprocessRaw(RGset)
mset<- mset[rowSums(failed) <= maxFail, ]
MSet.swan<- preprocessSWAN(RGset, mSet = mset)

## Matrix processed
## ----------------
betaV<- getBeta(MSet.swan)
colnames(betaV)<- paste('array', colnames(betaV), 'beta', sep= '.')
betaV<- data.frame(ID_REF= rownames(betaV), betaV)

detP<- data.frame(ID_REF= rownames(detP), detP)
matrixProcessed<- merge(detP, betaV, all.x= TRUE)
colOrder<- c('ID_REF',
    paste(rep(arrayNames, each= 2), c('beta', 'pval'), sep= '.'))
matrixProcessed<- matrixProcessed[, colOrder]
write.table(matrixProcessed, 'matrixProcessed.txt', col.names= TRUE,
    row.names= FALSE, sep= '\t', quote= FALSE, na= 'null')
stopifnot(nrow(matrixProcessed) == 485512)

## Matrix signal intensities
## -------------------------
unmeth<- getUnmeth(MSet.swan)
colnames(unmeth)<- paste('array', colnames(unmeth), 'U', sep= '.')
unmeth<- data.frame(ID_REF= rownames(unmeth), unmeth)
meth<- getMeth(MSet.swan)
colnames(meth)<- paste('array', colnames(meth), 'M', sep= '.')
meth<- data.frame(ID_REF= rownames(meth), meth)

matSigInt<- merge(detP, merge(meth, unmeth), all.x= TRUE)

arrayNames<- paste('array', paste(targets$Slide, targets$Array, sep= '_'),
    sep= '.')
colOrder<- c('ID_REF',
    paste(rep(arrayNames, each= 3), c('M', 'U', 'pval'), sep= '.'))
matSigInt<- matSigInt[, colOrder]
write.table(matSigInt, 'matrixSignalIntensities.txt', col.names= TRUE,
    row.names= FALSE, sep= '\t', quote= FALSE, na= 'null')

## Some checks
stopifnot(nrow(matSigInt) == 485512)
stopifnot(matSigInt$ID_REF == matrixProcessed$ID_REF)
```

2 References

[1] S F Altschul, W Gish, W Miller, E W Myers, and D J Lipman. Basic local alignment search tool. *Journal of molecular biology*, 215(3):403–10, October 1990.

[2] Martin J Aryee, Andrew E Jaffe, Hector Corrada-Bravo, Christine Ladd-Acosta, Andrew P Feinberg, Kasper D Hansen, and Rafael A Irizarry. Minfi: a flexible and comprehensive Bioconductor package for the analysis of Infinium DNA methylation microarrays. *Bioinformatics (Oxford, England)*, February 2014.

[3] Pankaj Chopra, Ligia A Papale, Andrew T White, Andrea Hatch, Ryan M Brown, Mark A Garthwaite, Patrick H Roseboom, Thaddeus G Golos, Stephen T Warren, and Reid S Alisch. Array-based assay detects genome-wide 5-mC and 5-hmC in the brains of humans, non-human primates, and mice. *BMC genomics*, 15(1):131, February 2014.

[4] G Dennis Jr., B T Sherman, D A Hosack, J Yang, W Gao, H C Lane, and R A Lempicki. DAVID: Database for Annotation, Visualization, and Integrated Discovery. *Genome Biol*, 4(5):P3, 2003.

[5] Jovana Maksimovic, Lavinia Gordon, and Alicia Oshlack. SWAN: Subset-quantile within array normalization for illumina infinium HumanMMaksimovic, J., Gordon, L., & Oshlack, A. (2012). SWAN: Subset-quantile within array normalization for illumina infinium HumanMethylation450 BeadChips. Genome biology, 13(6), R44. *Genome biology*, 13(6):R44, January 2012.

[6] A R Quinlan and I M Hall. BEDTools: a flexible suite of utilities for comparing genomic features. *Bioinformatics*, 26(6):841–842, 2010.

[7] Sabrina K Stewart, Tiffany J Morris, Paul Guilhamon, Harry Bulstrode, Martin Bachman, Shankar Balasubramanian, and Stephan Beck. oxBS-450K: A method for analysing hydroxymethylation using 450K BeadChips. *Methods (San Diego, Calif.)*, August 2014.

[8] Kai Wang, Mingyao Li, and Hakon Hakonarson. ANNOVAR: functional annotation of genetic variants from high-throughput sequencing data. *Nucleic acids research*, 38(16):e164, September 2010.

3 Supplementary Figures

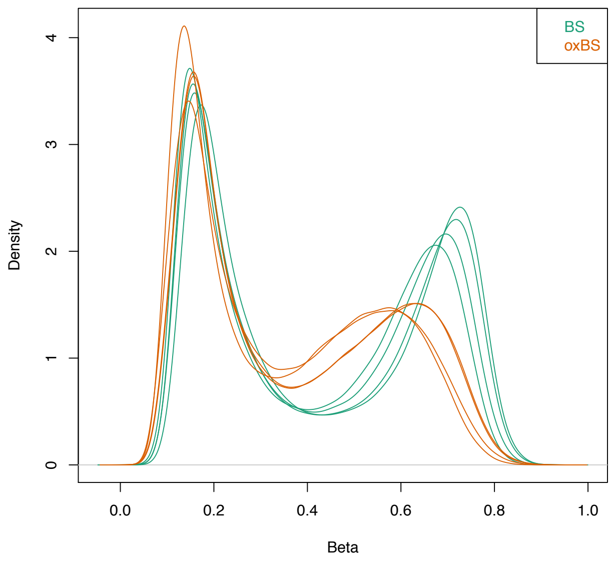

Figure S1: Density plot of beta values after having removed failed probes and after SWAN normalization.

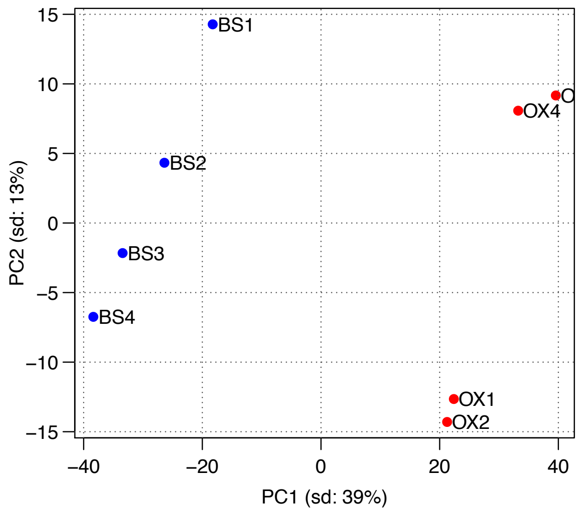

Figure S2: Principal components analysis based on probe beta values. In blue the "BS" arrays, in red the "oxBS" arrays.

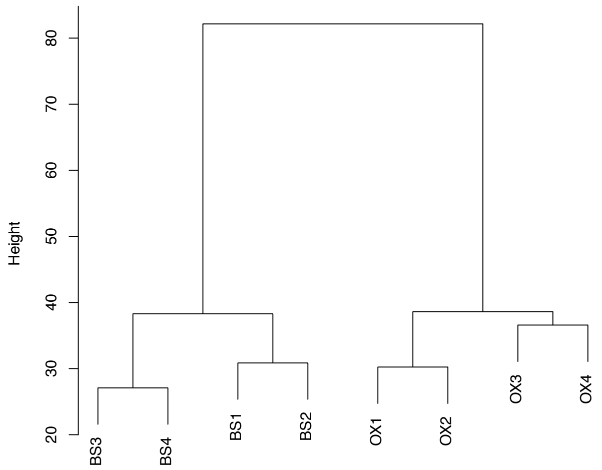

Figure S3: Hierarchical clustering of arrays on beta values.

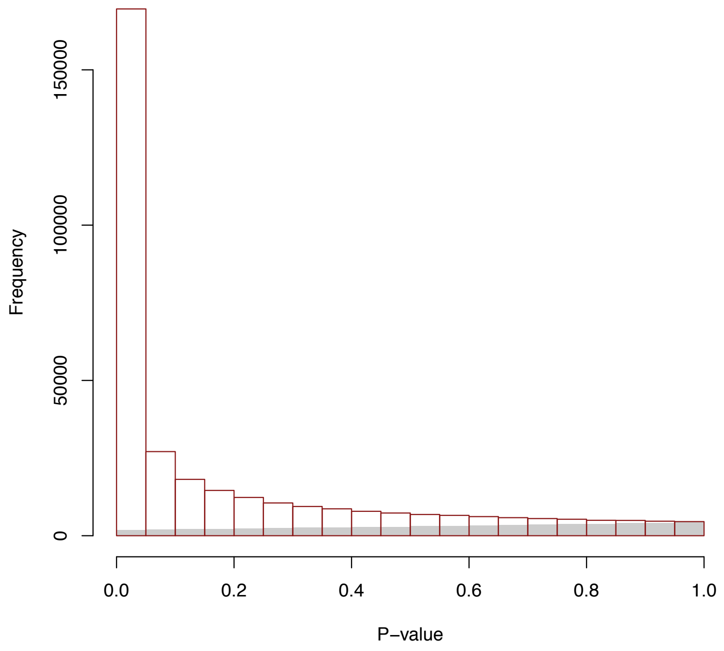

Figure S4: Histogram of pvalues for the evidence of BS - oxBS <0 (grey bars) and BS - oxBS >0 (red bars)

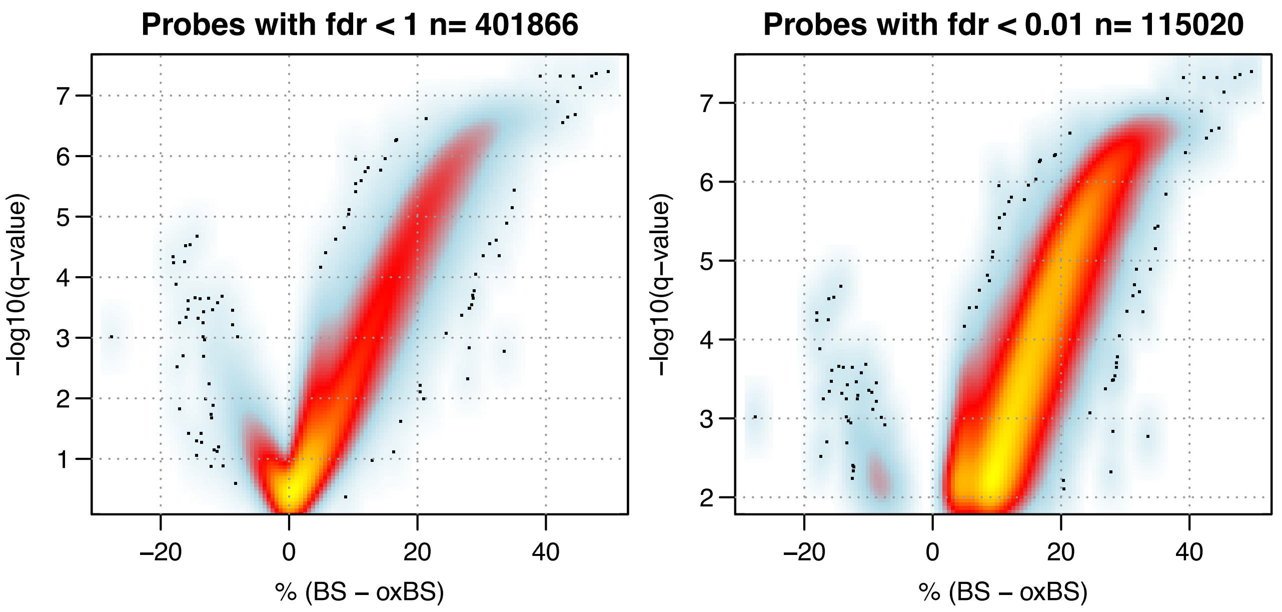

Figure S5: Probes differentially methylated between BS and oxBS arrays. Left: All the probes passing quality control (n= 401866). Right: Probes passing quality control and with FDR <0.01 for difference between BS and oxBS (n= 115020, n positive= 114734).

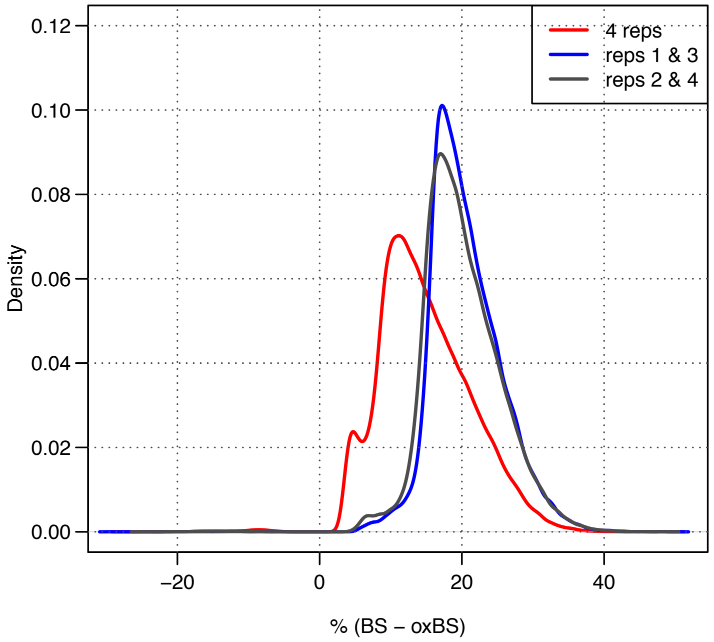

Figure S6: Effect size, *i.e.* significant difference in beta values between BS and oxBS, detected with four and two replicates.

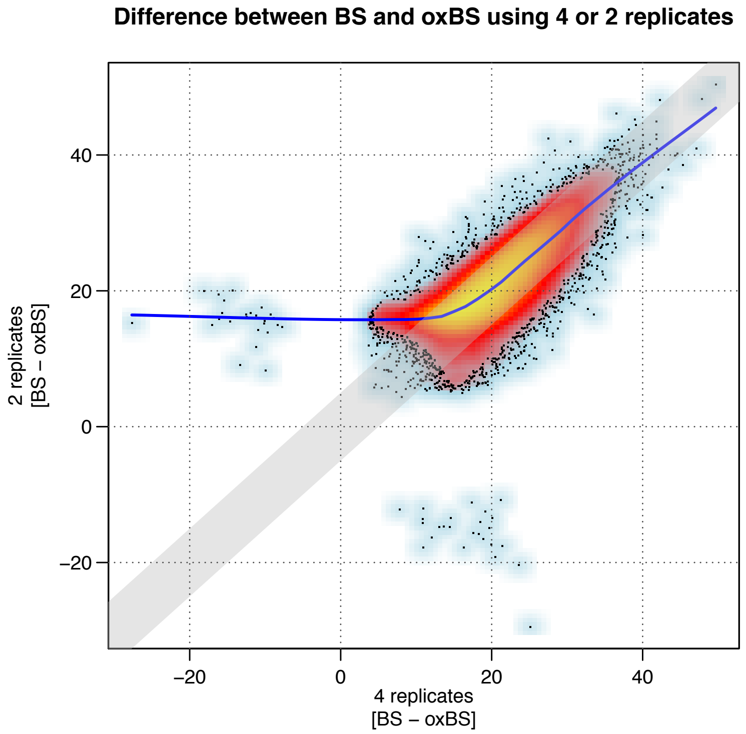

Figure S7: Difference between BS and oxBS beta values for probes detected significantly different in both the 4 replicates and in replicates 1 and 3. The shaded grey band marks the diagonal plus and minus 5, *i.e.* probes outside the grey band differ between 4 and 2 replicates by more than 5% in detected beta value.

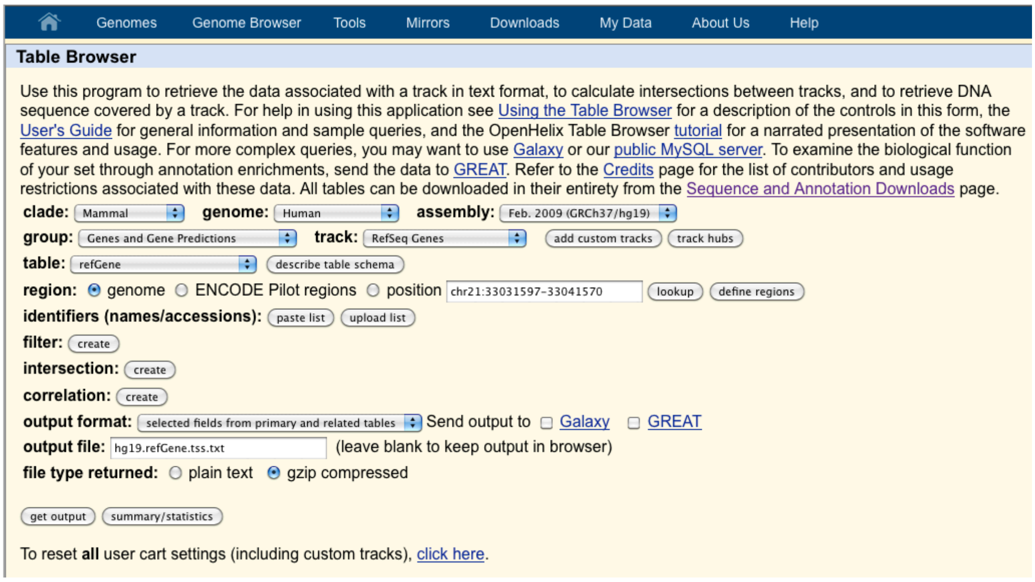

Figure S8: Screenshot from UCSC genome browser used to extract the positions of transcription start sites.

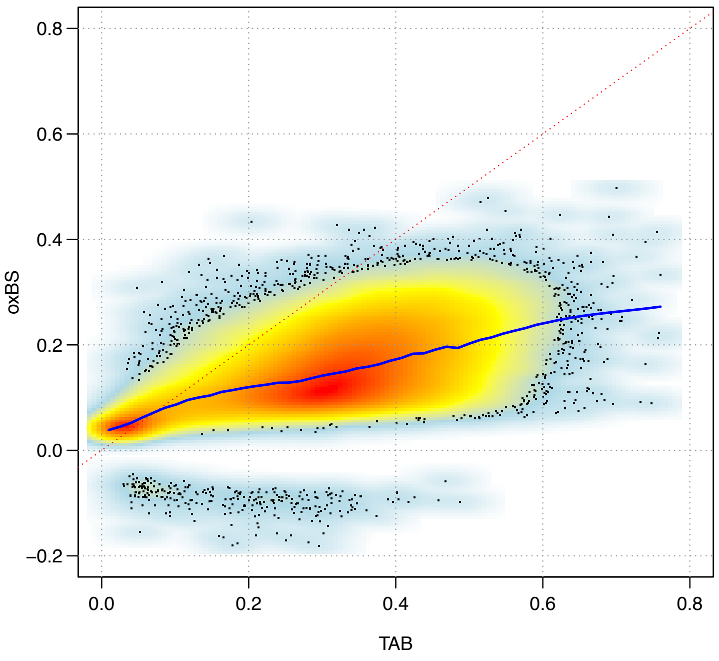

Figure S9: Comparison in estimated 5hmC between oxBS and TAB arrays [3]

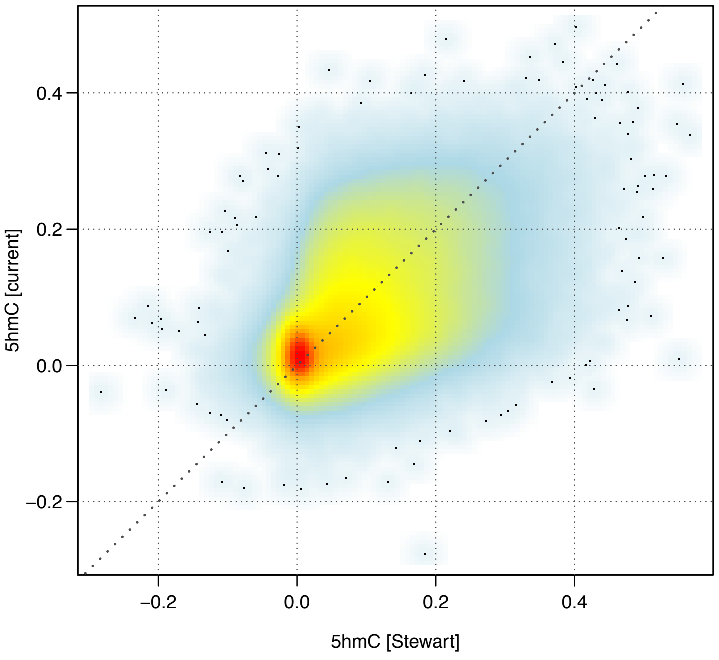

Figure S10: Comparison in estimated 5hmC between the current study and the 5hmC reported in Stewart *et al.* 2014 [7]

.

4 Appendix

4.1 Additional functions

```
 % -----------------------------------------------------------

aggSamp<- function(X, sampGroup) {
    "Aggregate values in matrix X by sampGroup
    Example:
        aggSamp(X= getBeta(MSet.swan), c('A', 'A', 'B', 'B'))
    "
    cnames<- sort(unique(sampGroup))
    aggMat<- matrix(data= NA, nrow= nrow(X), ncol= length(cnames))
    colnames(aggMat)<- cnames
    rownames(aggMat)<- rownames(X)
    for(i in 1:length(cnames)){
        xcol<- which(sampGroup == cnames[i])
        aggMat[, i]<- rowMeans(X[, xcol])
    }
    return(aggMat)
}

histPosNeg<- function(x, pval, posBorder= 'firebrick4',
freq= TRUE, xlab= 'P-value', ...){
    # Plot two histograms on the same plot for the pvalues of the
    # positive and negative scores
    # E.g. plot the histogram of pvalues for -ve % hmC and +ve % hmC.
    # x:
    #   Vector of percentages or in general a vetcor of positive and
    #   negative numbers.
    #   The actual value is not used, only the sign is used to separate pvalues.
    # pval:
    #   pvalues associated to x. The histograms are based on this vector.
    # ...:
    #   Futher args to pass to hist()
    # -------------------------------------------------------------------------
    if (length(x) != length(pval)){
        stop("Different length for x and pval vectors")
    }
    pneg<- pval[which(x <= 0)]
    ppos<- pval[which(x > 0)]

    ## y limits of histogrm
    if (freq == TRUE){
        f<- "counts"
    } else {
        f<- "density"
    }
    hneg<- max(hist(pneg, plot= FALSE)[[f]])
    hpos<- max(hist(ppos, plot= FALSE)[[f]])
    hlim<- c(0, max(c(hneg, hpos)))

    ## Plot
    hist(pneg, col= 'grey80', border= NA, ylim= hlim, freq= freq, xlab= xlab, ...)
    hist(ppos, col= 'transparent', border= posBorder, add= TRUE,
     freq= freq, xlab= xlab, ...)
}

makeTransparent<- function(col, alpha=0.5) {
  # Convert colour spcified as name or in other way to equivalent trasparent.
  # col:
  #    Vector of colours
  # alpha:
  #   Alpha transparency [0, 1]
  # See also (taken from)
  # http://stackoverflow.com/questions/8047668/transparent-equivalent-of-given-color
  #
  if(alpha<0 | alpha>1) stop("alpha must be between 0 and 1")

  alpha = floor(255*alpha)
  newColor = col2rgb(col= col, alpha=FALSE)
```

```
  .makeTransparent = function(col, alpha) {
    rgb(red=col[1], green=col[2], blue=col[3], alpha=alpha, maxColorValue=255)
  }

  newColor = apply(newColor, 2, .makeTransparent, alpha=alpha)

  return(newColor)
}


bedtools.intersect<-function(a, b, opt.string=""){
    # Functions to call bedtools tool from R.
    # Funtions are named bedtools.<tool name>.
    # This is an edit of http://zvfak.blogspot.co.uk/2011/02/calling-bedtools-from-r.html
    # ---------------------------------------------------------------------------

    #create temp files
    a.file<- tempfile(fileext = ".bed")
    b.file<- tempfile(fileext = ".bed")
    out   <- tempfile(fileext = ".bed")
    ori_scipen<- options('scipen')
    options(scipen= 99) # not to use scientific notation when writing out

    #write bed formatted dataframes to tempfile
    write.table(a, file= a.file, quote=F, sep="\t", col.names=F, row.names=F)
    write.table(b, file= b.file, quote=F, sep="\t", col.names=F, row.names=F)

    # create the command string and call the command using system()
    command<- paste('bedtools intersect', "-a", a.file, "-b", b.file , opt.string, ">",
        out, sep=" ")
    cat(command,"\n")
    try(system(command))

    res=read.table(out, header=F, stringsAsFactors= FALSE)
    unlink(a.file);unlink(b.file);unlink(out)
    options(scipen= ori_scipen)
    return(res)
}
```

4.2 qPCR primers

Table S3: Details of primers used for qPCR validation

Probe ID	Oligo name	Oligo sequence (5' to 3')	CpG position		Amplicon position	
CG13529101	FORWARD01	CCGAGCAGTACGAGGTCTAC	chr7	101845425	Start	101845283
CG13529101	REVERSE01	ACAGGCTGCGGGATAGATAC	chr7		End	101845481
CG19841423	FORWARD02	GCTCCTCGGAATGTGTTTGA	chr20	62366755	Start	62366686
CG19841423	REVERSE02	CGCTCGATCTTCTCCTCAGT	chr20		End	62366862
CG02351555	FORWARD03	GAACCCGCATGCTCCTATTG	chr19	1526463	Start	1526336
CG02351555	REVERSE03	GCCAAAGACTGTGGGTGAC	chr19		End	1526493
CG22237200	FORWARD04	GCTCACACTCCCTCTCGG	chr17	7555298	Start	7555221
CG22237200	REVERSE04	GATACAGACAACTTCCGCCG	chr17		End	7555430
CG12641434	FORWARD05	CCATGGGCAGTGTTTTCCTC	chr10	105992125	Start	105992000
CG12641434	REVERSE05	GCAGACCTTCCACCAATCTG	chr10		End	105992206
CG10061770	FORWARD06	GGAGCAAACTGTGTTGCCTC	chr16	68366844	Start	68366761
CG10061770	REVERSE06	AGTGAGCTGTGTTCGCATCA	chr16		End	68366973
CG16959747	FORWARD07	CACAATCTGGGCTTGAAGGG	chr12	7276714	Start	7276637
CG16959747	REVERSE07	AACGCCCTCTTTCTCTCTCC	chr12		End	7276814
CG12882907	FORWARD08	AACGACTAGCAGGGAGATCC	chr12	96428593	Start	96428508
CG12882907	REVERSE08	TCTTCAGGCCCCAGACTTTT	chr12		End	96428704
CG18918390	FORWARD09	CTCCGCCACTAACCCTTGA	chr10	21605038	Start	21604987
CG18918390	REVERSE09	TCTGTCCTTGGAAGATGTGTGA	chr10		End	21605087
CG19644590	FORWARD10	TGGACGTTTACTTCTTGCTCC	chr19	1937198	Start	1937172
CG19644590	REVERSE10	CGTGTCTTCTTCCTGCTGTC	chr19		End	1937341
CG12131862	FORWARD11	GGGCTTCAGTGACAGAGACT	chr1	203613877	Start	203613828
CG12131862	REVERSE11	GGAGAGTGAAAGGTGGAGCT	chr1		End	203613978
CG08133755	FORWARD12	GGGTCAGAGGTCTCCCAAAG	chr11	19792080	Start	19791982
CG08133755	REVERSE12	CCATGAATCCAAGGGTTGCC	chr11		End	19792137
CG02667291	FORWARD13	GGACCAAGACCCATGTTTTACC	chr19	897142	Start	896996
CG02667291	REVERSE13	ATCGTTGAGCTTTTCTGGCC	chr19		End	897163
CG13685679	FORWARD14	CTGCCTCATTTTCCCACGG	chr17	79231688	Start	79231558
CG13685679	REVERSE14	CGTTCGTACCTGCTCTCTCT	chr17		End	79231787
CG27553486	FORWARD15	AGTGCACTTGTCAGCCTGA	chr7	930964	Start	930834
CG27553486	REVERSE15	GCATTTCCAGATAGTGCGGG	chr7		End	931035
CG26875137	FORWARD16	GATGTGAGGGGTGAGTAGTCA	chr12	53738046	Start	53737912
CG26875137	REVERSE16	CAGGTCCTCAGATACTGCCC	chr12		End	53738085
CG09828580	FORWARD17	TAAAGCTGTCCTCGTCGTCA	chr10	103868007	Start	103867950
CG09828580	REVERSE17	TTGCCCCGATTATGTTTGGG	chr10		End	103868118
CG10837846	FORWARD18	AGACTCACACGAACTCCAGG	chr14	91758641	Start	91758617
CG10837846	REVERSE18	AATATCGCCAGCAGAGGTTG	chr14		End	91758779
CG09832245	FORWARD19	GTGAGAGTGGGCTGCAAATC	chr16	85494611	Start	85494474
CG09832245	REVERSE19	CTTCCCACTCCAGAGCTCAC	chr16		End	85494643
CG01272627	FORWARD20	GTTTGAGACCACTGTGCTGG	chr5	180659531	Start	180659461
CG01272627	REVERSE20	GAATGGTGCAAAGGGGATGG	chr5		End	180659655
CG06805880	FORWARD21	CCCTCCGCACCTCATTCTTA	chr17	27401144	Start	27401111
CG06805880	REVERSE21	CTCTCCATCACAGCTGCATC	chr17		End	27401293
CG07141452	FORWARD22	GGTACTCCTGCCTTGCTGA	chr20	3775639	Start	3775529
CG07141452	REVERSE22	CTAAATGCCTTCCAGCCCC	chr20		End	3775712
CG14429457	FORWARD24	ACCAGCTAACGTCGAGATGT	chr10	665304	Start	665268
CG14429457	REVERSE24	CTGTGCTCCCGATATTCTGC	chr10		End	665444
CG08321942	FORWARD25	ACACACAAAGGAGAGGGGTT	chr19	34310625	Start	34310504
CG08321942	REVERSE25	GCCCTGAAGACCTAGCTCTT	chr19		End	34310664
CG16613029	FORWARD26	TGGCACCAAATCCACTTGAC	chr16	9052762	Start	9052632
CG16613029	REVERSE26	AAAGGGCCCACAGTAACCAT	chr16		End	9052811
CG22117062	FORWARD28	CCCCAAGACGACCTCTGG	chr4	2794231	Start	2794207
CG22117062	REVERSE28	CAAGACAGGAGCAGGGGAG	chr4		End	2794366
CG18524262	FORWARD29	GGGTGACTGGTTGATTTCGG	chr18	55862111	Start	55862019
CG18524262	REVERSE29	TAGCTTCGTGACATCCAGCA	chr18		End	55862215
