# Supplementary figures and images for: Accurate Measurement of 5-Methylcytosine and 5-Hydroxymethylcytosine in Human Cerebellum DNA by Oxidative Bisulfite on an Array (OxBS-Array)
Source: PLoS One. 2015 Feb 23;10(2):e0118202. doi: 10.1371/journal.pone.0118202 (PMC4338296; doi:10.1371/journal.pone.0118202)

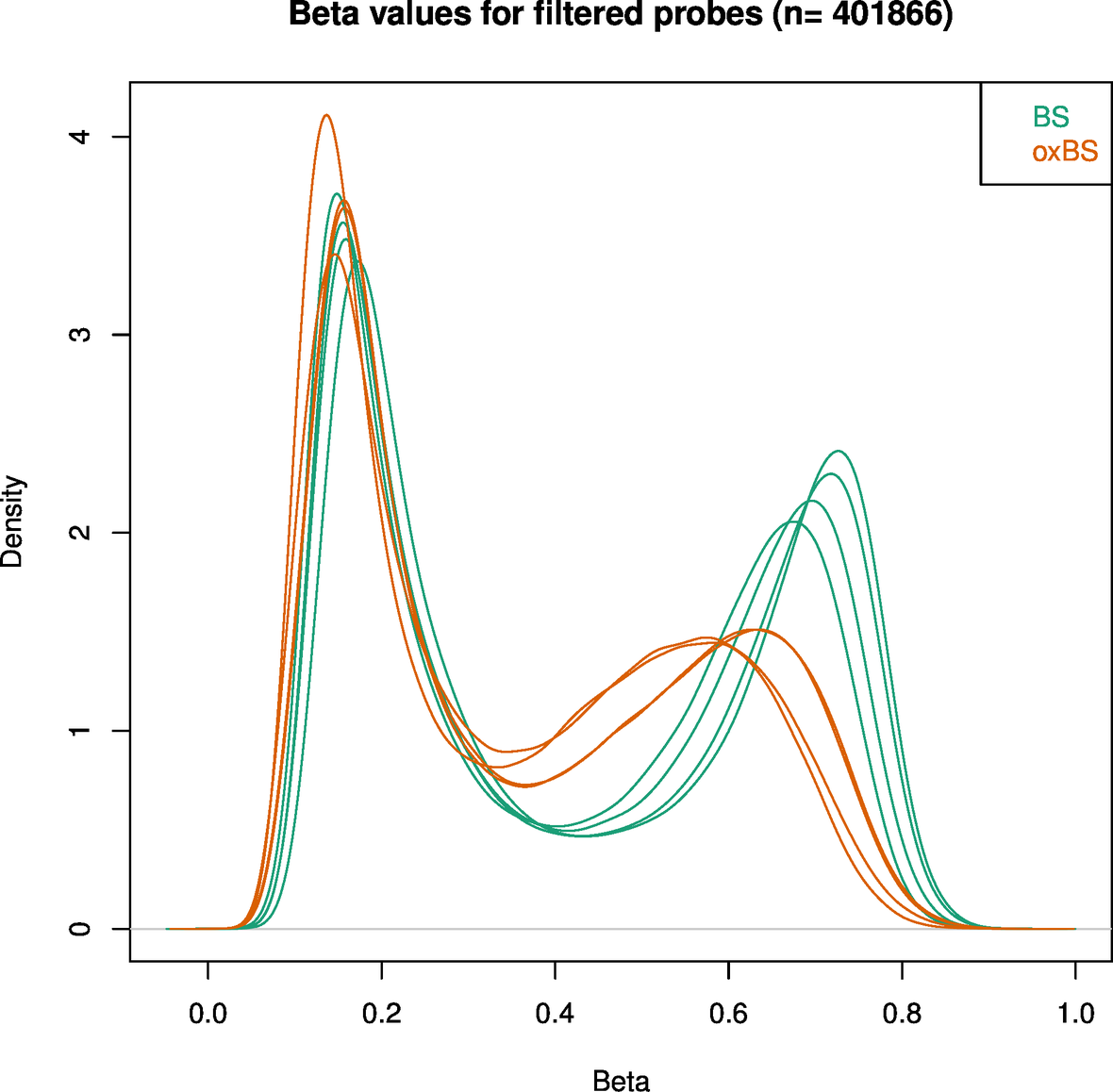

Supplement: S1 Fig — (TIFF) [file pone.0118202.s002.tiff]

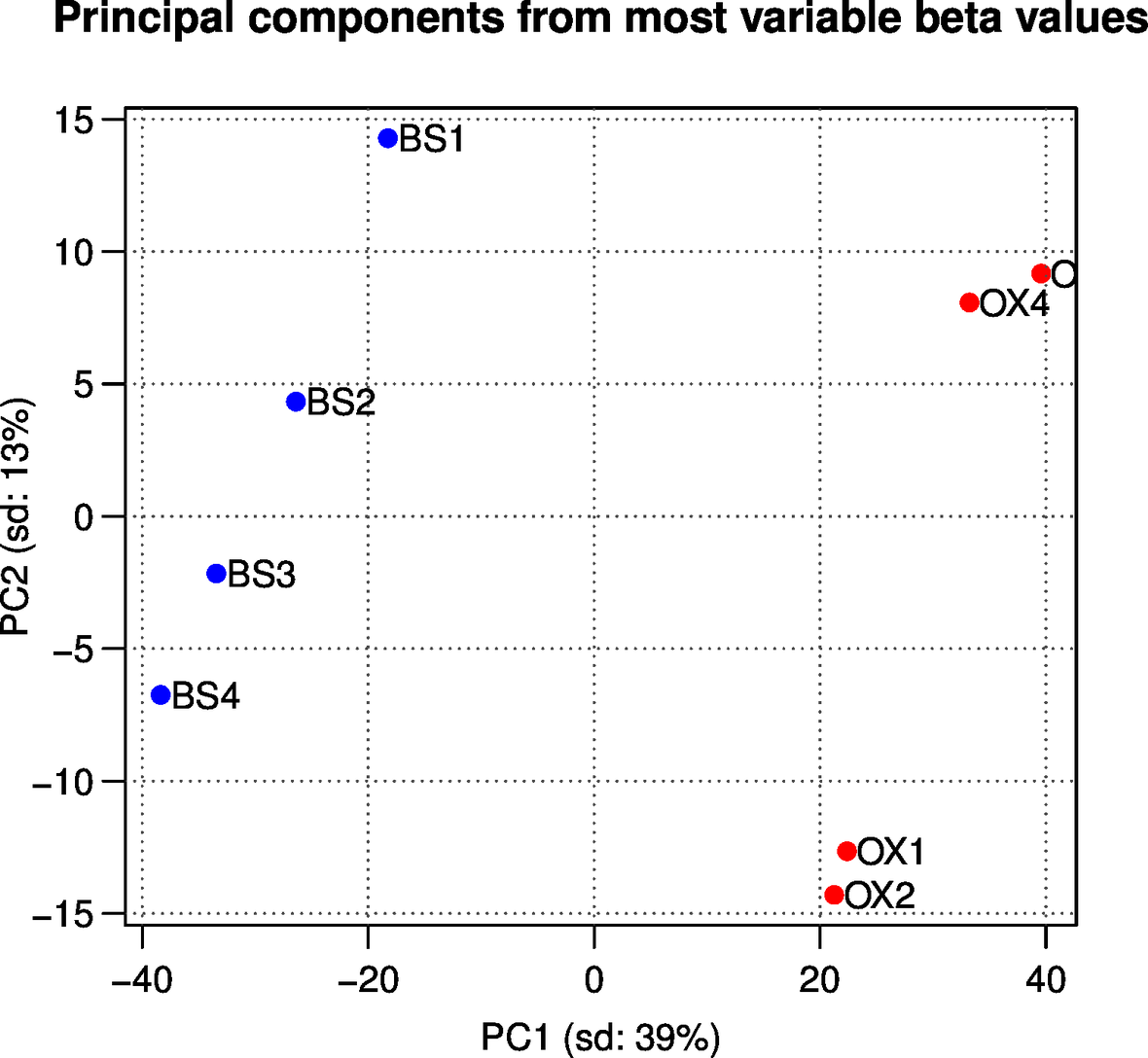

Supplement: S2 Fig — In blue the “BS” arrays, in red the “oxBS” arrays. (TIFF) [file pone.0118202.s003.tiff]

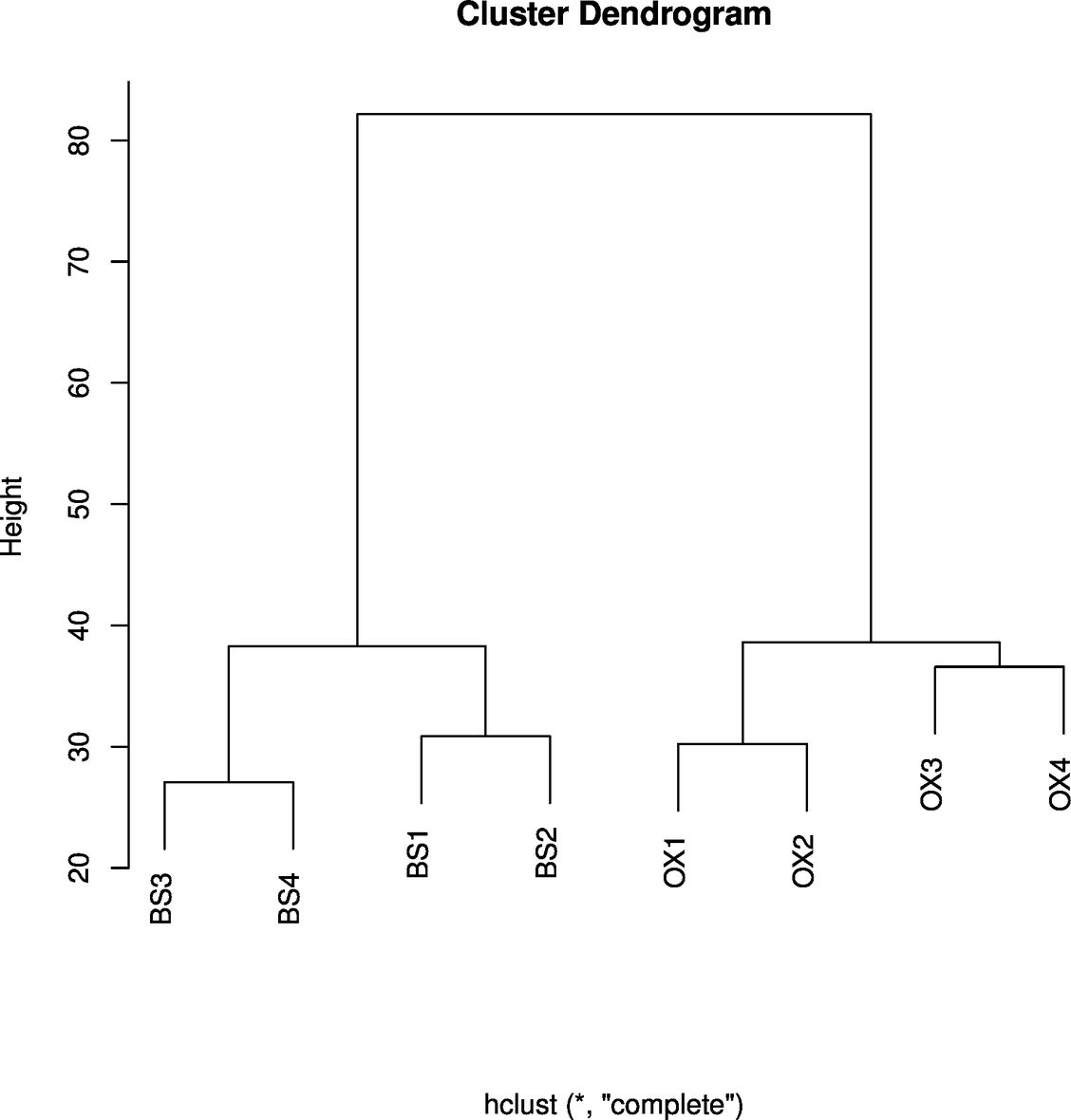

Supplement: S3 Fig — (TIFF) [file pone.0118202.s004.tiff]

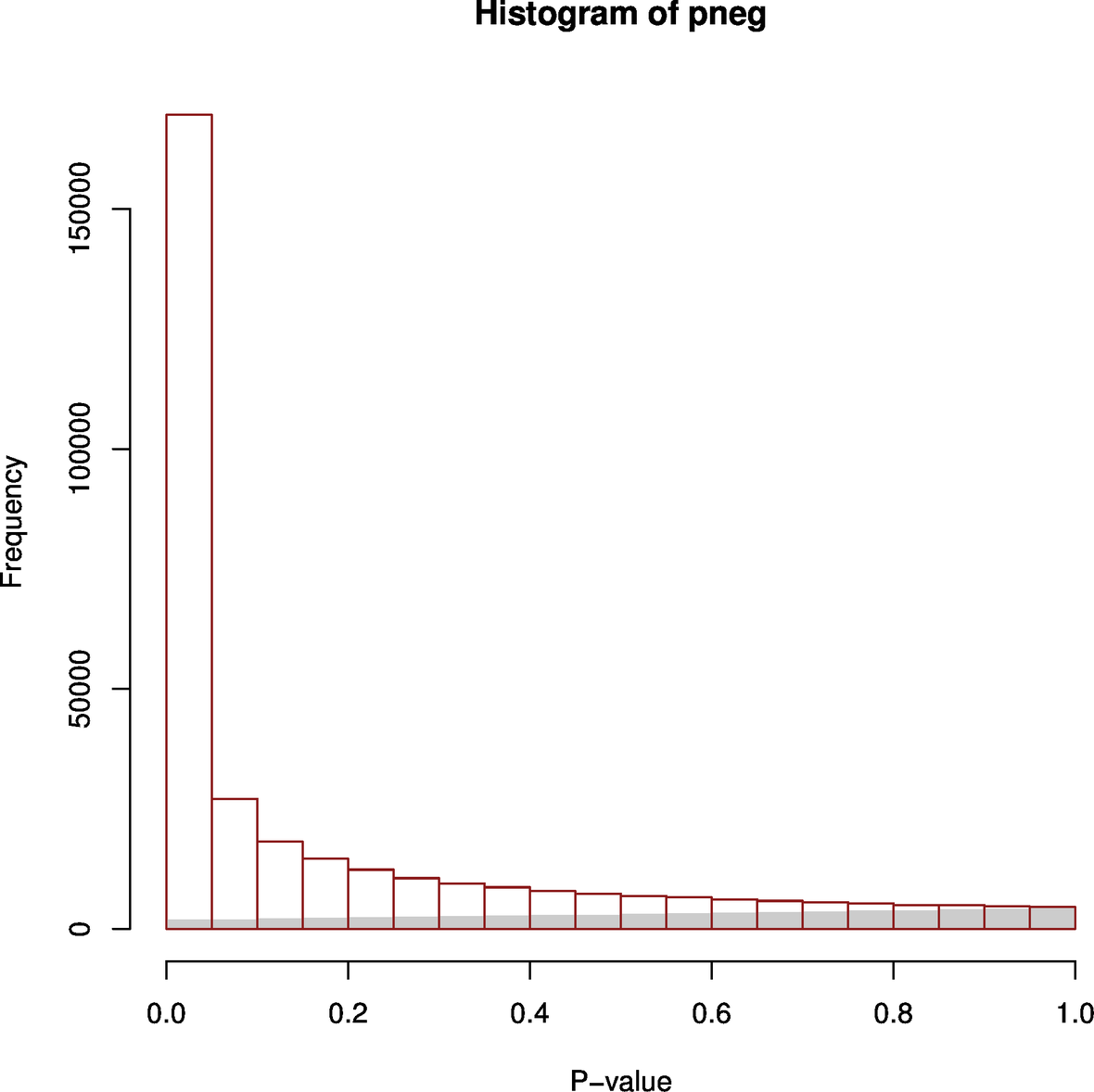

Supplement: S4 Fig — (TIFF) [file pone.0118202.s005.tiff]

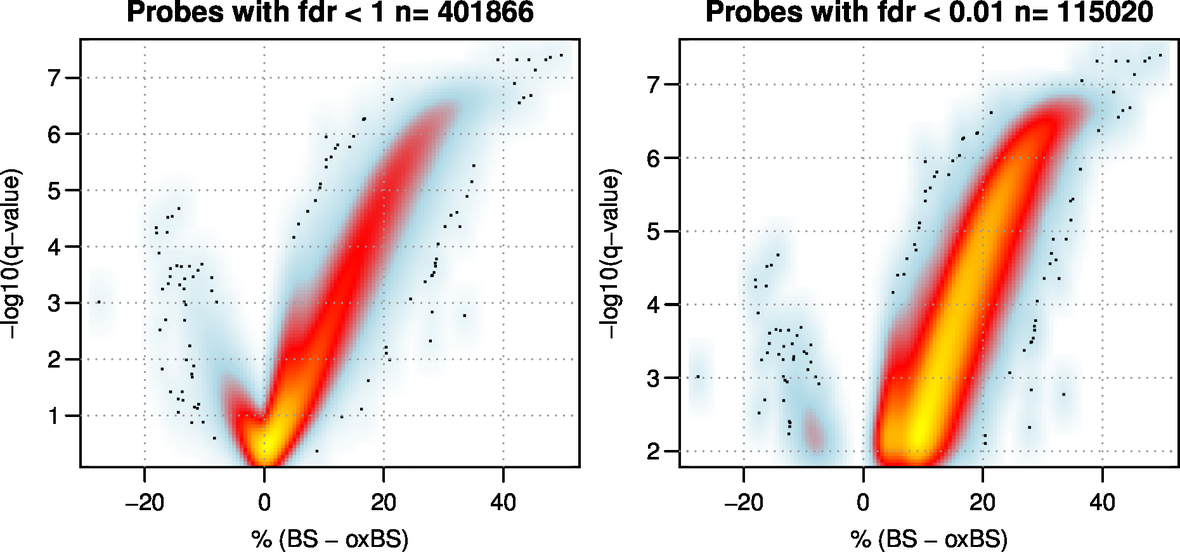

Supplement: S5 Fig — Left: All the probes passing quality control (n = 401866). Right: Probes passing quality control and with FDR <0.01 for difference between BS and oxBS (n = 115020, n positive = 114734). (TIFF) [file pone.0118202.s006.tiff]

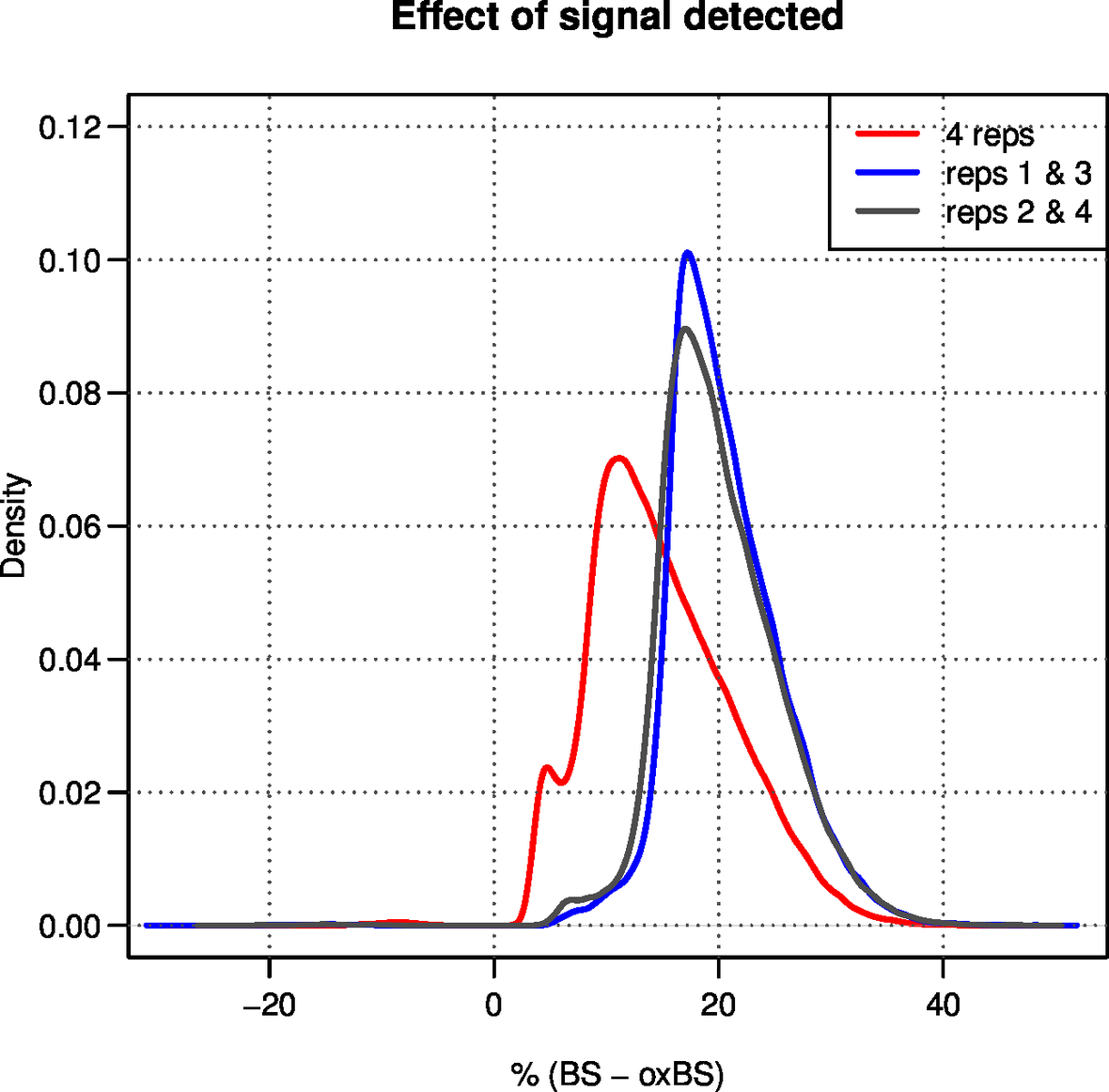

Supplement: S6 Fig — (TIFF) [file pone.0118202.s007.tiff]

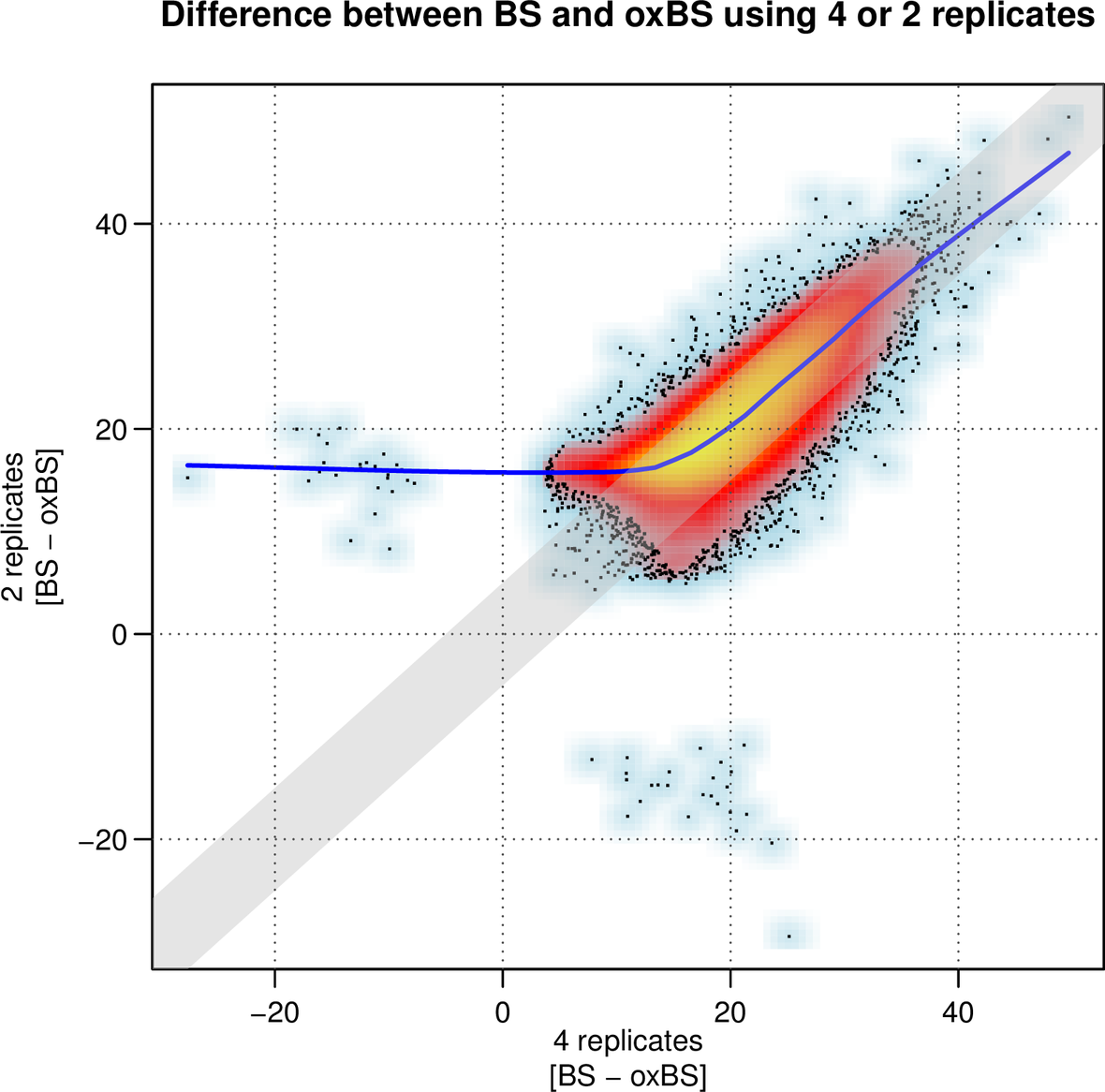

Supplement: S7 Fig — The shaded grey band marks the diagonal plus and minus 5, i.e. probes outside the grey band differ between 4 and 2 replicates by more than 5% in detected beta value. (TIFF) [file pone.0118202.s008.tiff]

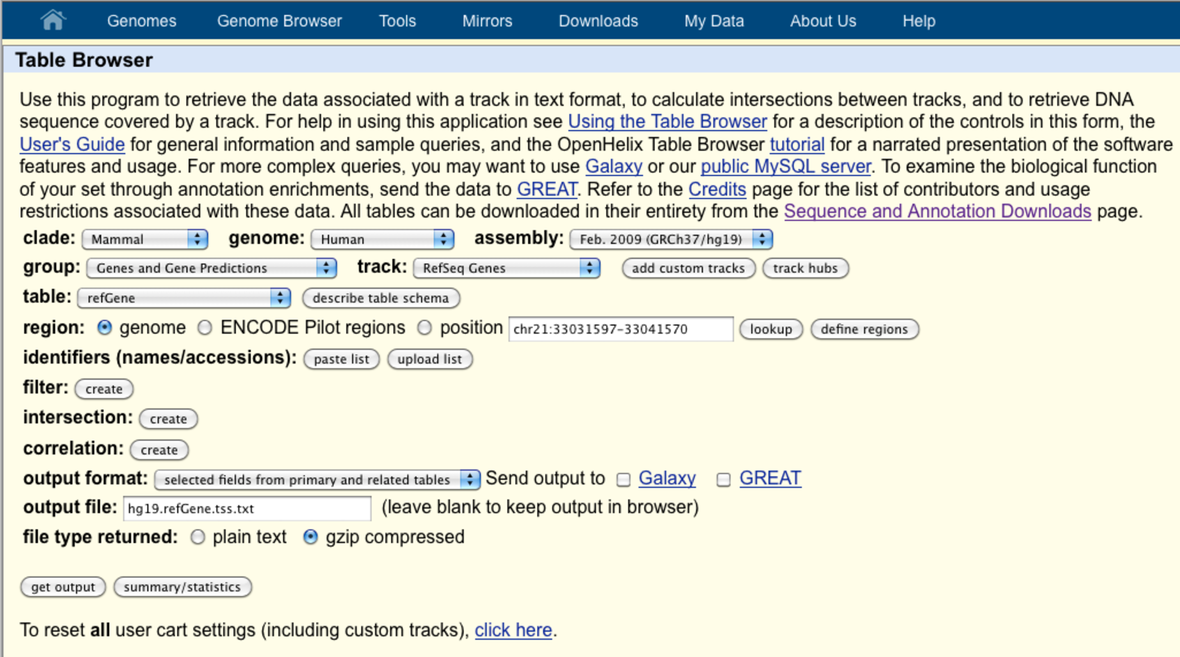

Supplement: S8 Fig — (TIFF) [file pone.0118202.s009.tiff]

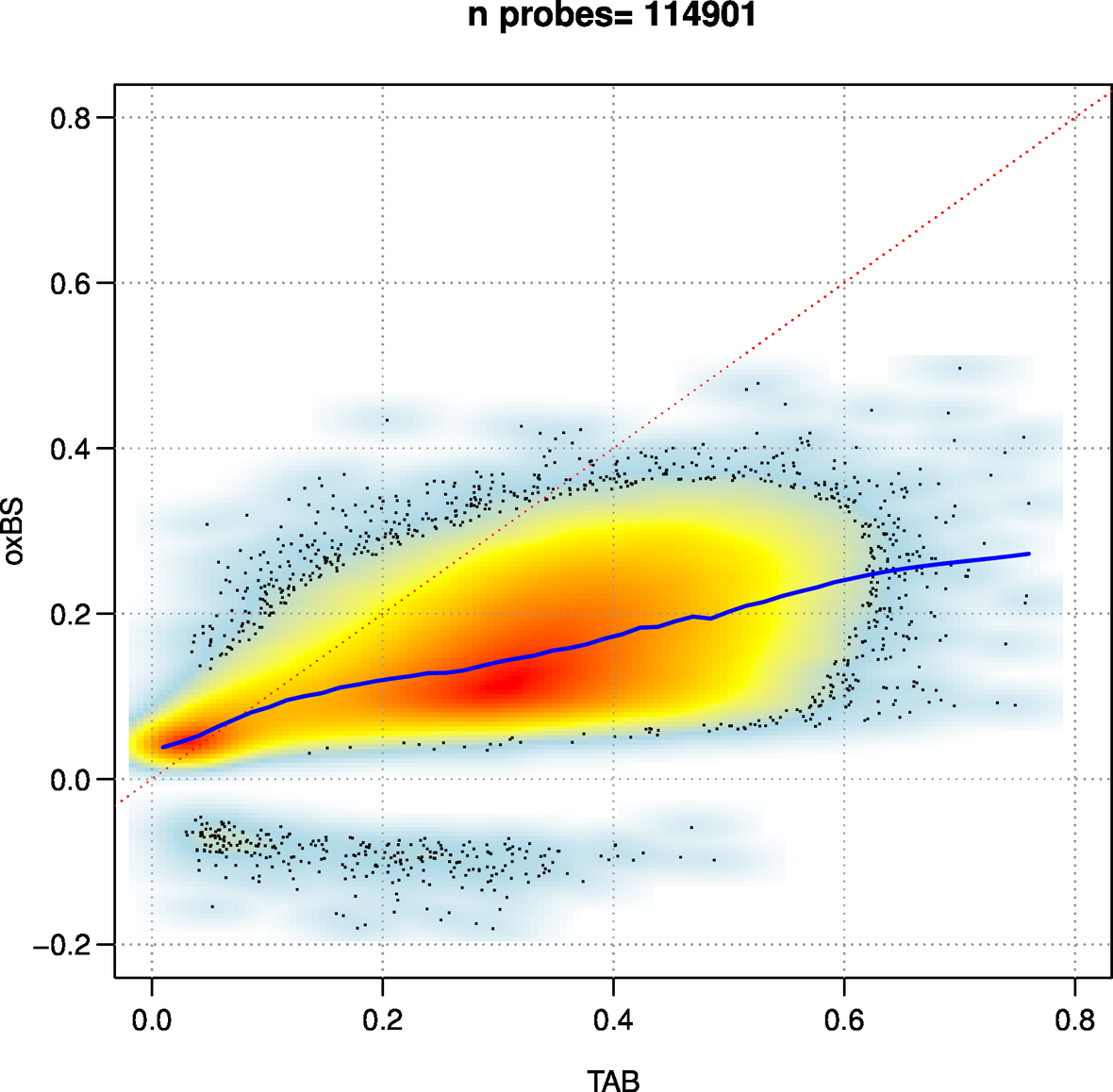

Supplement: S9 Fig — (TIFF) [file pone.0118202.s010.tiff]

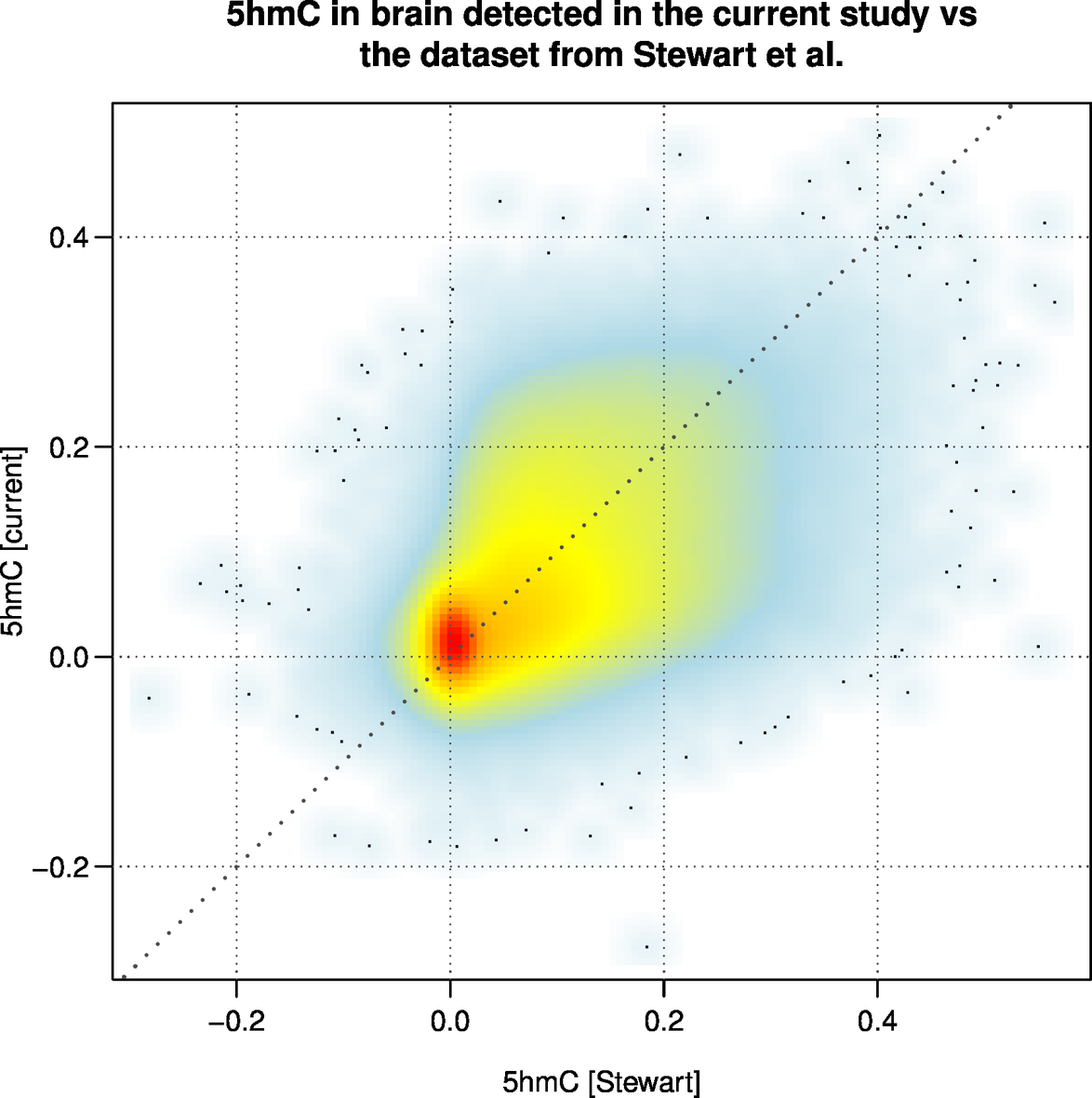

Supplement: S10 Fig — (TIFF) [file pone.0118202.s011.tiff]
