# Supplementary material for: Accurate Measurement of 5-Methylcytosine and 5-Hydroxymethylcytosine in Human Cerebellum DNA by Oxidative Bisulfite on an Array (OxBS-Array)
Source: PLoS One. 2015 Feb 23;10(2):e0118202. doi: 10.1371/journal.pone.0118202 (PMC4338296; doi:10.1371/journal.pone.0118202)
Supplement: S1 Table — (PDF) [file pone.0118202.s012.pdf]

	Sample_Name	Sample_Group	Basename
1	BS1	Bisulfite	9373551079_R01C01
2	BS2	Bisulfite	9373551079_R02C01
3	BS3	Bisulfite	9373551079_R03C01
4	BS4	Bisulfite	9373551079_R04C01
5	OX1	Oxidation Bisulfite	9373551079_R05C01
6	OX2	Oxidation Bisulfite	9373551079_R06C01
7	OX3	Oxidation Bisulfite	9373551079_R01C02
8	OX4	Oxidation Bisulfite	9373551079_R02C02
