# Supplementary material for: Accurate Measurement of 5-Methylcytosine and 5-Hydroxymethylcytosine in Human Cerebellum DNA by Oxidative Bisulfite on an Array (OxBS-Array)
Source: PLoS One. 2015 Feb 23;10(2):e0118202. doi: 10.1371/journal.pone.0118202 (PMC4338296; doi:10.1371/journal.pone.0118202)
Supplement: S2 Table — (PDF) [file pone.0118202.s013.pdf]

	probe_id	% qPCR	% 450k
1	cg02351555	0	-10
2	cg19841423	0	-10
3	cg22237200	0	-6
4	cg12641434	1	3
5	cg13529101	1	-12
6	cg16959747	8	10
7	cg18918390	8	10
8	cg10061770	9	10
9	cg19644590	9	10
10	cg12882907	10	10
11	cg02667291	18	20
12	cg12131862	19	20
13	cg27553486	19	20
14	cg13685679	20	20
15	cg08133755	21	20
16	cg09828580	27	30
17	cg26875137	27	30
18	cg06805880	28	30
19	cg01272627	29	30
20	cg07141452	30	30
21	cg09832245	30	30
22	cg10837846	30	30
23	cg18524262	30	40
24	cg14429457	34	36
25	cg08321942	35	36
26	cg22117062	36	38
27	cg16613029	37	37
