# Supplementary material for: Accurate Measurement of 5-Methylcytosine and 5-Hydroxymethylcytosine in Human Cerebellum DNA by Oxidative Bisulfite on an Array (OxBS-Array)
Source: PLoS One. 2015 Feb 23;10(2):e0118202. doi: 10.1371/journal.pone.0118202 (PMC4338296; doi:10.1371/journal.pone.0118202)
Supplement: S3 Table — (PDF) [file pone.0118202.s014.pdf]

Probe ID	Oligo name	Oligo sequence (5′ to 3′)	CpG position		Amplicon position	
CG13529101	FORWARD01	CCGAGCAGTACGAGGTCTAC	chr7	101845425	Start	101845283
CG13529101	REVERSE01	ACAGGCTGCGGGATAGATAC	chr7		End	101845481
CG19841423	FORWARD02	GCTCCTCGGAATGTGTTTGA	chr20	62366755	Start	62366686
CG19841423	REVERSE02	CGCTCGATCTTCTCCTCAGT	chr20		End	62366862
CG02351555	FORWARD03	GAACCCGCATGCTCCTATTG	chr19	1526463	Start	1526336
CG02351555	REVERSE03	GCCAAAGACTGTGGGTGAC	chr19		End	1526493
CG22237200	FORWARD04	GCTCACACTCCCTCTCGG	chr17	7555298	Start	7555221
CG22237200	REVERSE04	GATACAGACAACTTCCGCCG	chr17		End	7555430
CG12641434	FORWARD05	CCATGGGCAGTGTTTTCCTC	chr10	105992125	Start	105992000
CG12641434	REVERSE05	GCAGACCTTCCACCAATCTG	chr10		End	105992206
CG10061770	FORWARD06	GGAGCAAACTGTGTTGCCTC	chr16	68366844	Start	68366761
CG10061770	REVERSE06	AGTGAGCTGTGTTCGCATCA	chr16		End	68366973
CG16959747	FORWARD07	CACAATCTGGGCTTGAAGGG	chr12	7276714	Start	7276637
CG16959747	REVERSE07	AACGCCCTCTTTCTCTCTCC	chr12		End	7276814
CG12882907	FORWARD08	AACGACTAGCAGGGAGATCC	chr12	96428593	Start	96428508
CG12882907	REVERSE08	TCTTCAGGCCCCAGACTTTT	chr12		End	96428704
CG18918390	FORWARD09	CTCCGCCACTAACCCTTGA	chr10	21605038	Start	21604987
CG18918390	REVERSE09	TCTGTCCTTGGAAGATGTGTGA	chr10		End	21605087
CG19644590	FORWARD10	TGGACGTTTACTTCTTGCTCC	chr19	1937198	Start	1937172
CG19644590	REVERSE10	CGTGTCTTCTTCCTGCTGTC	chr19		End	1937341
CG12131862	FORWARD11	GGGCTTCAGTGACAGAGACT	chr1	203613877	Start	203613828
CG12131862	REVERSE11	GGAGAGTGAAAGGTGGAGCT	chr1		End	203613978
CG08133755	FORWARD12	GGGTCAGAGGTCTCCCAAAG	chr11	19792080	Start	19791982
CG08133755	REVERSE12	CCATGAATCCAAGGGTTGCC	chr11		End	19792137
CG02667291	FORWARD13	GGACCAAGACCCATGTTTTACC	chr19	897142	Start	896996
CG02667291	REVERSE13	ATCGTTGAGCTTTTCTGGCC	chr19		End	897163
CG13685679	FORWARD14	CTGCCTCATTTTCCCACGG	chr17	79231688	Start	79231558
CG13685679	REVERSE14	CGTTCGTACCTGCTCTCTCT	chr17		End	79231787
CG27553486	FORWARD15	AGTGCACTTGTCAGCCTGA	chr7	930964	Start	930834
CG27553486	REVERSE15	GCATTTCCAGATAGTGCGGG	chr7		End	931035
CG26875137	FORWARD16	GATGTGAGGGGTGAGTAGTCA	chr12	53738046	Start	53737912
CG26875137	REVERSE16	CAGGTCCTCAGATACTGCCC	chr12		End	53738085
CG09828580	FORWARD17	TAAAGCTGTCCTCGTCGTCA	chr10	103868007	Start	103867950
CG09828580	REVERSE17	TTGCCCCGATTATGTTTGGG	chr10		End	103868118
CG10837846	FORWARD18	AGACTCACACGAACTCCAGG	chr14	91758641	Start	91758617
CG10837846	REVERSE18	AATATCGCCAGCAGAGGTTG	chr14		End	91758779
CG09832245	FORWARD19	GTGAGAGTGGGCTGCAAATC	chr16	85494611	Start	85494474
CG09832245	REVERSE19	CTTCCCACTCCAGAGCTCAC	chr16		End	85494643
CG01272627	FORWARD20	GTTTGAGACCACTGTGCTGG	chr5	180659531	Start	180659461
CG01272627	REVERSE20	GAATGGTGCAAAGGGGATGG	chr5		End	180659655
CG06805880	FORWARD21	CCCTCCGCACCTCATTCTTA	chr17	27401144	Start	27401111
CG06805880	REVERSE21	CTCTCCATCACAGCTGCATC	chr17		End	27401293
CG07141452	FORWARD22	GGTACTCCTGCCTTGCTGA	chr20	3775639	Start	3775529
CG07141452	REVERSE22	CTAAATGCCTTCCAGCCCC	chr20		End	3775712
CG14429457	FORWARD24	ACCAGCTAACGTCGAGATGT	chr10	665304	Start	665268
CG14429457	REVERSE24	CTGTGCTCCCGATATTCTGC	chr10		End	665444
CG08321942	FORWARD25	ACACACAAAGGAGAGGGGTT	chr19	34310625	Start	34310504
CG08321942	REVERSE25	GCCCTGAAGACCTAGCTCTT	chr19		End	34310664
CG16613029	FORWARD26	TGGCACCAAATCCACTTGAC	chr16	9052762	Start	9052632
CG16613029	REVERSE26	AAAGGGCCCACAGTAACCAT	chr16		End	9052811
CG22117062	FORWARD28	CCCCAAGACGACCTCTGG	chr4	2794231	Start	2794207
CG22117062	REVERSE28	CAAGACAGGAGCAGGGGAG	chr4		End	2794366
CG18524262	FORWARD29	GGGTGACTGGTTGATTTCGG	chr18	55862111	Start	55862019
CG18524262	REVERSE29	TAGCTTCGTGACATCCAGCA	chr18		End	55862215
